# Technological Ecosystems in Care and Assistance: A Systematic Literature Review

**DOI:** 10.3390/s19030708

**Published:** 2019-02-09

**Authors:** Samuel Marcos-Pablos, Francisco José García-Peñalvo

**Affiliations:** GRIAL Research Group, Research Institute for Educational Sciences, University of Salamanca, 37008 Salamanca, Spain; fgarcia@usal.es

**Keywords:** systematic literature review, technological ecosystems, care and assistance technologies

## Abstract

Applying the concepts of technological ecosystems to the care and assistance domain is an emerging field that has gained interest during the last years, as they allow to describe the complex relationships between actors in a technologically boosted care domain. In that context, this paper presents a systematic review and mapping of the literature to identify, analyse and classify the published research carried out to provide care and assistance services under a technological ecosystems’ perspective. Thirty-seven papers were identified in the literature as relevant and analysed in detail (between 2003–2018). The main findings show that it is indeed an emerging field, as few of the found ecosystem proposals have been developed in the real world nor have they been tested with real users. In addition, a lot of research to date reports the proposal of platform-centric architectures developed over existing platforms not specifically developed for care and services provision. Employed sensor technologies for providing services have very diverse natures depending on the intended services to be provided. However, many of these technologies do not take into account medical standards. The degree of the ecosystems’ openness to adding new devices greatly depends on the approach followed, such as the type of middleware considered. Thus, there is still much work to be done in order to equate other more established ecosystems such as business or software ecosystems.

## 1. Introduction

Providing care and assistance services to people with special care needs (older people, disabled people, people with chronic health issues) is a complex task. Different actors such as health organizations, health and care workers, regulators and governing bodies, and even IT solution providers may be involved with the provision of a single service. According to the World Health Organization [1], many existing health systems still manage health issues in a disconnected and fragmented way, and there is still a lack of coordination across care providers, stakeholders, organizations and settings as well as in the timing of the care provided.

In this sense, although recent advances in sensor technologies have spurred the care and assistance field, expanding the possibilities of providing better and more reliable technological solutions for assisting people with disabilities [2], many of the current developments still have not evolved from an end user-centric perspective. Many studies have denoted the need to change this approach towards a broader perspective in the development of technology for care provision [3,4,5], taking into account the different stakeholders, application goals, business models, etc. in the design, development and implementation process of not only sensors, but also the different related technological components involved in providing care and assistance services (platforms, protocols, ontologies, etc.).

During the last decade, however, there has been an increasing interest in the development of different types of solutions that allow the interaction between the different actors in the health and care sector, in order to deliver integrated and age-friendly care and assistance services [6,7,8,9,10,11]. This interest has been spurred by governing authorities, as the development of such ecosystems is beneficiary due to three main factors: affordability, as health services tend to be expensive; regulatory, searching for regional, national and international standard compliance in order to provide safe health and care services; availability, in order to provide specialized services in remote regions within a particular country.

Thus, the current trend in IT solution providers (encompassing more than just the health sector) is to move from fragmented services to progressively more integrated services, which are likely to be provided by multiple stakeholders through well-elaborated collaboration mechanisms [3].

As such, the field of technological ecosystems has emerged as the study of the complex interaction between technological devices (ambient sensors, wearables, medical devices, etc.) and extensible technological frameworks and architectures on the one hand, and organizations, users, customers, developers, and businesses on the other [3,12].

The definition of a technological ecosystem has evolved throughout the years (see [13] for a comprehensive review of the evolution of the term), but it comes from the concept of Biological Ecosystems first defined by British ecologist Arthur Tansley in 1935 to denote the physical and biological components of an environment when considered in relation to each other as a unit [14]. Technological ecosystems can indeed be compared to biological ecosystems from the perspective of resource management and biodiversity, as they allow relationships between software projects, products, communities, and organizations [15]. Some approaches consider technological ecosystems from a business-oriented point of view as a network of actors, organizations, and companies [16,17], while others are more oriented to the ecosystem architecture, technical aspects and communities of users of the involved technological platforms [18,19]. A recent paper from Iyawa et al. [20] defines a digital health innovation ecosystem as “a network of digital health communities consisting of interconnected, interrelated and interdependent digital health species, including healthcare stakeholders, healthcare institutions and digital healthcare devices situated in a digital health environment, who adopt the best-demonstrated practices that have been proven to be successful, and implementation of those practices through the use of information and communication technologies to monitor and improve the well-being and health of patients, to empower patients in the management of their health and that of their families”.

However, even though the technological ecosystem concept has gained importance within the research community in recent years [20,21], there is still a lack of consensus on what a technological care and assistance ecosystem is, which components of this type of ecosystem are relevant in the care and assistance domain, what architectures are the most appropriate for developing this type of ecosystem, or even how the community of users interacts with each other. Until recently, most research and developments in care and assistance services have been focused on the development of techno-centric and/or isolated services (e.g., faint/fall detection [22], seniors’ motion monitoring [23], physiological monitoring [24,25], cardiac early warning systems [26] measuring ambient air quality [27], smart appliances [28], etc.). However, the importance of the role of communities and other forms of collaborative networks involving all stakeholders, operating as an ecosystem, is being recognized [3,29].

Taking the above into account, from the point of view of the sensor research and development community it is important to have a comprehensive vision of the whole care and assistance ecosystem (beyond the mere development of sensors or care related devices), in order to obtain an overview of the needs of the rest of the ecosystem actors involved (dependent people, formal and informal caregivers, doctors, authorities, etc.) and identify the strengths and weaknesses of the research presented to date, which in turn will point out the possible future research directions along with their potential applications, stakeholders and market. In this sense, there have been different systematic reviews related to the Ambient Assisted Living (AAL) domain such as [2,4,30,31] which provide a nice review of different components, technologies or attributes that conform to the care and assistance ecosystem. However, none of them are focused on the whole technological ecosystem concept.

As care and assistance technological ecosystems are an open and new research area, which involves evolution capabilities and actors’ inter-relationships and added value, going beyond specific solutions for health or AAL, in this paper we want to provide an overview of the research work reported in the field by means of a Systematic Literature Review (SLR). To sum it up, the purpose of this systematic literature review is to obtain an overview of the research reported in the field of technological ecosystems aimed to provide services to people with special care needs.

The rest of the paper is organized as follows: Section 2 identifies the existing literature reviews related to the current topic. Section 3 describes the research questions, the protocol followed for selecting the studies and the process for extracting the relevant data, including the mapping and research questions. Section 4 presents the synthesis results of the data extracted. Section 5 offers a discussion of the data extracted in order to answer the formulated research questions. Section 7 adds a more critical discussion regarding the research gaps, challenges and opportunities that can be extracted from the revised literature. Conclusions are presented in Section 8.

## 2. Related Work

Following the recommendations of [32,33], prior to undertaking a systematic review, researchers should ensure that a systematic review is necessary, identifying and reviewing any existing systematic reviews of the phenomenon of interest against appropriate evaluation criteria. Thus, as an initial step of our systematic review, we followed a similar approach as the one taken in [13]. To do so, we followed a protocol analogous to the main search of our systematic review, which will be further explained in the following sections. We performed an initial literature search on the following electronic databases: Web of Science, Scopus, IEEE Xplore, ACM Digital Library and Springer Link, broadening the scope of the results so they included any literature review (also called secondary studies [32]) in the field of digital, software or technological ecosystems. The search was performed based on the article title, abstract and keywords and the employed query string was:

(“digital ecosystem*” OR “software ecosystem*” OR “tech* ecosystem*”) AND (“state of the art” OR “SLR” OR “review” OR “systematic mapping” OR “systematic literature review”).

As a result of this search, we obtained a total of 1335 results, from which we removed all duplicates across the databases and stored them in a spreadsheet (http://bit.ly/2Pfr9xT). Next, we analyzed the title and abstract of the resultant papers and applied the following inclusion criteria:

1. The papers were written in English AND
2. The papers were published in peer reviewed Journals, Books, Conferences or Workshops AND
3. The papers performed a systematic review of the existing literature AND
4. The systematic review focused on digital/technological/software ecosystems AND
5. The technological ecosystems are within the care, assistance or health sector AND

The paper’s compliance with the inclusion criteria was verified by the reviewers following the next steps:

1. To speed up the screening process, the criteria 1 and 2 were first considered and we took into account the paper language, the publication venue, and if the paper abstract was available.
2. Then the reviewers manually applied criteria 3 and 4, reading through titles and abstracts of the downloaded studies in order to assess if the resultant papers performed a review focused on digital, technological or software ecosystems. A paper fulfilled these criteria if: the title or abstract contained the term ecosystem, and the title or abstract suggested that the authors could have performed a systematic review related to the topic.
  In the few cases where the title and abstract were not sufficient to make a decision, the authors quickly assessed the entire content of the paper. In these cases, reviews were taken into account if they at least outlined the protocol followed for obtaining relevant literature from the scientific databases. On the other hand, they were considered to be related technological, software or digital ecosystems if the paper addressed the notion of ecosystem on either its business, organizational or software structure. That is, rather than just considering dedicated solutions, platforms or system of systems, the paper should consider the evolutionary characteristics of the ecosystems and at least refer to the actors involved. Papers were included in the next stages only if both reviewers agreed they fulfilled the inclusion criteria.
  After these first two rounds the resultant candidate papers were labeled as “Candidate for full paper reading” in the spreadsheet (http://bit.ly/2Pfr9xT), obtaining a total of 24 candidate papers. It has to be noted that also during the assessment of this criteria three cases were detected with missing abstracts that could be candidates for full paper reading based just on the paper title, so they were also included in the next step.
3. The entire content of those 21 papers was assessed in order to see if all or at least some of the research or mapping questions in these systematic reviews were indeed related to the health or care sector. Papers that were compliant with this criteria were labeled as “Related to healthcare” in the spreadsheet.

As a result of this last step, we finally obtained three secondary studies:

In [20] they present a scoping review aimed at identifying the scope and range of digital health, innovation and digital ecosystems literature in developed and developing countries, and propose guidelines for implementing digital health innovation ecosystems.

In [34] they perform a review on the existing literature on digital health innovation ecosystems from which they try to identify the main components of three different concepts: digital health, innovation in health, and digital ecosystems. They also propose a definition of the term *digital health innovation ecosystems*, to finally present a conceptual map of the related terms previously selected.

Ref. [35] is a two pages length paper presented in the Iranian Journal of Public Health which claims to perform a review of technological ecosystems in hospital management and health care informatics. However, the scarce information given by the authors makes it difficult to extract proper conclusions or assess the validity of their study.

From this first evaluation of the literature, we can conclude that, although there exist some secondary studies in the literature that refer to related software ecosystems [12,13], mobile ecosystems [36] or business ecosystems [37], there is a lack of systematic reviews or secondary studies related to the selected topic, and so conducting a systematic mapping review about technological ecosystems in the care and assistance sector is justified.

In addition, this first step served us as a pilot search from which we extracted the following conclusions:

- the term “ecosystem” if included as a single search keyword produces a great amount of results related to biological sciences but which are not related to the technological field.
- “health” is a keyword that mainly refers to the concept of the well-functioning of an ecosystem. Hence, the term “ecosystem health” is employed in many studies as the ability of the ecosystem to endure and remain variable and productive over time [12], and could mislead to some results not related to the care and assistance sectors.
- it is necessary to broaden the search with the selected search string as there is a limited number of publications related to ecosystems in healthcare.

## 3. Methods

The following sections describe the process undertaken, which follows the recommendations of Kitchenham [32] and Pedersen [33] regarding the methodologies for conducting systematic literature reviews and mapping studies. The final goal is to identify gaps and research opportunities in the area, by formulating a set of relevant research questions and identifying and evaluating the related studies in the literature. Thus, the following sections describe the main activities proposed by Kitchenham: planning, conducting and reporting the study. In order to define the process to follow during the different stages, we identified the main aspects that were relevant in order to build the review protocol. This section lays out the results of said planning, including the research questions, the inclusion/exclusion criteria, the quality criteria and the followed search strategy.

### 3.1. Review Scope

As part of the planning stage, we have formulated three main research questions which have been broken down into a set of several sub-questions that will drive the review method. In order to contextualize, frame and answer these questions, and explain our review scope, we started by defining the PICOC items proposed by Petticrew and Roberts [38]. The PICOC acronym stands for:

- Population (P): the target group for the investigation. As we are interested in two areas simultaneously—technological ecosystems and care—, our population is, in fact, a conjunction of those two populations.
- Intervention (I): specifies the investigation aspects or issues of interest for the researchers. In our case the design, implementations, components, performance evaluations, etc. of technological ecosystems proposals.
- Comparison (C): refers to aspect of the investigation with which the intervention is being compared to. No comparison intervention has been planned in our study.
- Outcomes (O): the effect of the intervention. We seek ecosystem proposals related to care and assistance as well as real implementations
- Context (C): the setting or environment of the investigation. In our case, they are those environments related to care and assistance (in healthcare, industry, academia, etc.).

Taking the PICOC model into account, we translated our research goals into the following main research questions (RQs):

RQ1 ***How are care and assistance technological ecosystems considered in the literature?*** During the last years many different fields have employed the ecosystem metaphor to different extents. When compared to those other communities, we want to explore if the care and assistance ecosystems have any particularity in terms of their definition, scope, evolution over time, etc.

RQ2 ***What are the main distinctive characteristics of this type of ecosystems?*** As described in our PICOC model, our population is comprised by two very different research fields: one is related to care and assistance whereas the other has to do mainly with technology. In terms of the first field, we are interested in investigating who are the principal actors participating in the care and assistance ecosystems, how does each actor contribute to the ecosystem, what are the barriers that complicate or hinder those contributions and what is the maturity of the proposals found in the literature.

RQ3 ***How are sensor technologies integrated in the care ecosystem proposals?*** In ecology terms, an ecosystem is a unit of interdependent organisms which share the same habitat [16], so it would be interesting to investigate the level of integration, collaboration and information exchange between the two populations inside the ecosystem: the assistive and the technical components. From a technological point of view, we are interested in knowing which type of architectures are proposed for this type of ecosystems, what type of sensor technologies are employed for providing services within the ecosystem, how they are integrated in the ecosystem architecture and how they relate to the care and assistance scope.

#### Mapping Questions

The above main questions have been broken down into a set of mapping questions (MQs) that will help to explore and analyze the current state of the art in the field. The considered mapping questions were:

Regarding RQ1:

- MQ1.1 What definitions of care and assistance ecosystems are given in the literature?
- MQ1.2 How has the research field of care and assistance ecosystems evolved over time? I.e., number of publications per year.
- MQ1.3 What are the main publication venues?
- MQ1.4 Who are the most active authors in the area?

Regarding RQ2:

- MQ2.1 What is the ecosystem goal in terms of care and assistance?
- MQ2.2 What care and assistance services are provided?
- MQ2.3 Who are the main actors and what are their value propositions?
- MQ2.4 How can actors enhance the ecosystem functionalities?
- MQ2.5 What is the ecosystem maturity? I.e., have proposals been deployed in real world conditions or only conceptual architectures?

Regarding RQ3:

- MQ3.1 What type of ecosystem architectures have been proposed in the literature?
- MQ3.2 What modelling notation and guidelines have been employed for describing the ecosystem architecture?
- MQ3.3 What sensor technologies are employed for providing care and assistance services?
- MQ3.4 How are sensors integrated in those architectures?
- MQ3.5 How do sensor technologies take into account the care and assistance environment? I.e., do they follow medical standards?
- MQ3.6 Are there complementary training actions considered in the ecosystem? E.g., in terms of technology usage, devices integration, etc.

### 3.2. Database Selection

After defining the research and mapping questions (RQs, MQs), we selected the information sources of the papers to be included in the search process. The search was conducted in the following electronic databases: Web of Science and Scopus. The databases were chosen accordingly to the following requirements:

- The database is available for us through our institution.
- The database is capable of using logical expressions or a similar mechanism.
- The database allows full-length searches or searches only in specific fields of the works.
- The database allows additional filtering options such as publication year or publication language.
- The database is one of the most relevant in the research areas of interest in this review process: engineering, computer science, and care and assistance technologies.

### 3.3. Search String

As stated in [39], building the search string is one of the main challenges when performing an SLR. In order to answer the proposed research questions, a Systematic Literature Review needs to be capable of capturing all possible results that relate to the topic of interest. With that aim, the employed search string must contain key terms related to that topic, so that the obtained results are relevant. In [32] they suggest identifying the main search string terms from the PICOC model (Population, Intervention, Comparison, Outcomes and Context) as well as from the research questions. However, as we are interested in two populations simultaneously, we employed a similar approach as the one followed by [13] and built our search string from our population dimension: technological ecosystems on the one hand, and care and assistance services for dependent people on the other.

From here, a common approach for building the search string is to manually choose the search terms, taking the researchers’ knowledge of the field as a basis, and refining the chosen terms by performing pilot searches until a search string that produces adequate results is found. However, as stated in Section 2, when piloting our search we obtained a great variability in both the total number of results as well as in the amount of results related to the field of interest (Table 1 shows an example of different pilot searches). For that reason, in order to build the search string we employed a mixed approach. On the one hand, we selected a set of search terms for both population dimensions based on our previous knowledge and the performed pilot searches. On the other hand, we developed an iterative methodology for search string construction along with a set of decision support tools that help in building the search string by finding appropriate key terms related to the topic of interest. This methodology and tools are described in depth in our paper [40].

As such, our final search string was:

(ecosystem OR SECO OR platform) AND (elderly OR “older people” OR care OR “Ambient Assisted Living” OR AAL OR dependent OR dependant)

### 3.4. Inclusion and Exclusion Criteria

Due to the broad scope of our review population and therefore of our search string, we made use of the databases search tools in order to delimit the number of obtained results. We limited the search to the engineering and computer sciences fields, as we were interested in ecosystems of technological nature. In addition, we limited the results from 2003 to date, because it can be considered the year where the concept of business ecosystem adopted the digital component which later originated other concepts such as technological ecosystem or software ecosystem [13].

Finally, making use of a set of tools in the form of python scripts we developed for our paper which are available in (http://bit.ly/2PwL37v), we are able to compute the similarity (in terms of cosine distance) between two document abstracts. By using this approach, we have computed the distance between all the abstracts in the corpus of results and three selected prototype abstracts that we considered that resemble the topic of interest (see Table 2). We have then qualitatively selected a minimum distance as inclusion criteria from which we considered that the abstracts are similar to the considered prototypes and thus their associated publications should be included in our review.

**Figure 1:**
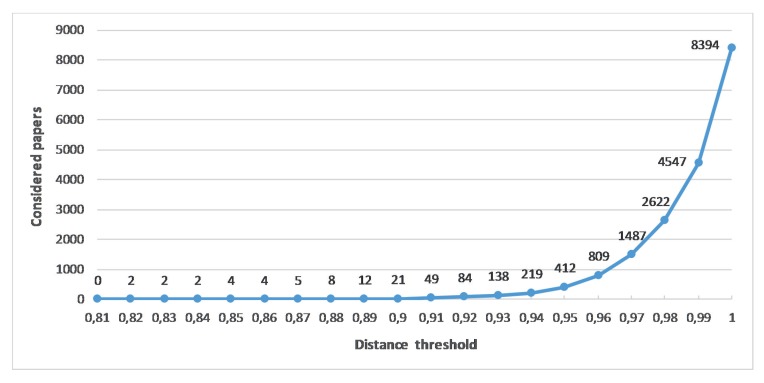
Number of papers to be considered depending on the distance threshold.

The selection of this parameter has been qualitatively done, using a mixed approach that involves the set of tools and procedures as described in [40]. Figure 1 shows the number of papers from our final search that would be included versus this parameter.

On the one hand, an SVM classifier has been trained with a group of papers that resulted from one of our piloted searches. These papers have been manually labeled as relevant/not relevant for the review scope, and the classifier is used to allow a raw classification of the new papers that may be related to the review. On the other hand, the cosine distance is used to sort the papers in terms of their similarity to the abstract prototypes, providing an indication of the number of papers labeled as relevant that would be included in the review if a particular threshold is selected. Finally, a visual inspection approach was performed by the researchers (that is, the results are analyzed by reviewing the titles and abstracts of randomly selected papers) to assess the correctness of the obtained results. Following this methodology, we considered that a threshold of 0.96 was adequate, both for producing a manageable number of relevant studies and for providing an adequate threshold to eliminate many that were not relevant according to the classifier.

It has to be noted that the set of tools used are not intended to fully automate the process of paper selection; they are decision support tools. The final decision is always in the hands of the researchers’ criteria which are reflected in the quality criteria described in Section 3.5.

Taking the above into account, our final inclusion criteria were:

- Papers should address technological ecosystems related to care and assistance technologies.
- Only papers published between 2003 and 2018.
- Papers published in peer reviewed Journals, Books, Conferences or Workshops.
- Papers written in English.
- Papers that have a document body that is more than three pages long.
- Papers’ abstract cosine distance to any of the three prototype abstracts is less than 0.96.

### 3.5. Quality Assessment Criteria

Assessing the quality of a primary study is a challenging task while performing a systematic literature. Thus, as shown in the guidelines proposed by Kitchenham and Charters [32], a quality checklist is suggested to be applied to the candidate studies to assess them and avoid subjectivity. These checklists are useful to assist in the paper selection process. The customized quality assessment checklist developed is based on the checklist suggested in [32]. It consists of a series of questions to be answered from a fast reading of the paper content, where the answer to each of the questions is labeled as Yes/Partially/No and given a score of 0/0.5/1 respectively. The Yes/Partially/No values stand for: Yes = information is explicitly defined/evaluated; Partially = information is implicit/stated; No = information is not inferable, except from the question “Is the ecosystem implemented in the real world?”

In this case we refer to the maturity level of the ecosystem, ranging from just a proposal to having been implemented in the real world. That is, we evaluate the closeness of the ecosystem to be running real conditions. If the ecosystem is just a proposal, it is labelled as No. If it has been piloted to some extent it is labelled as Partially, and if it has been deployed and running it is labelled as Yes.

Table 3 shows the checklist developed for the current systematic review.

### 3.6. Review and Mapping Protocol

The protocol followed for the review process consisted of the following main steps:

1. The search was conducted in the databases indicated in Section 3.2 and using the query string described in Section 3.3 with the database filtering options enumerated in Section 3.4. All the results were collected in .csv format. The downloaded list of candidate papers included for each document: title, abstract, authors, publication year, publication venue, etc.
2. The inclusion criteria was then applied to the downloaded list of candidate papers. In order to reduce the number of results to be manually analyzed, the automated abstract similarity inclusion criteria was first applied, restricting the list of candidate papers to 809. Results from this first selection are shown in (http://bit.ly/2BpCxV0).
3. The resultant papers were then assessed for complying the rest of the inclusion criteria of Section 3.2 based on their title and abstract. In those cases where the title and abstract were not sufficient to make a decision, the authors quickly assessed the entire content of the paper. The resultant candidate papers were added to another sheet of the spreadsheet document (http://bit.ly/2Mkfujv).
4. The papers were then read in detail and analyzed based on the quality assessment checklist described in Table 3) and the results were collected in another spreadsheet (http://bit.ly/2xXUxRu). In addition, during this assessment we considered papers collected within the references of those ones that potentially could be interesting for the review and mapping process, and the same quality assessment was performed over them. This last step provided the authors with another 5 papers to review.

**Figure 2:**
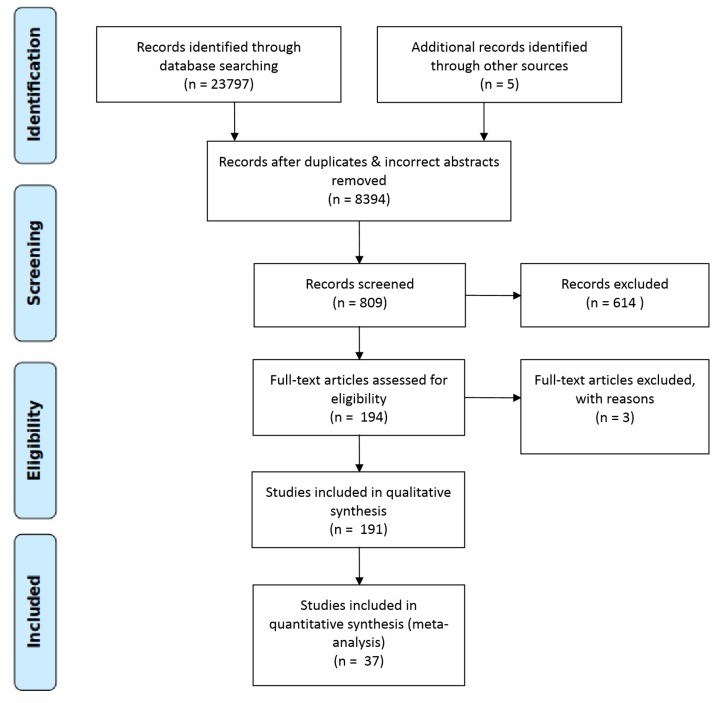
Steps and results of review and mapping process. Reported as proposed in the PRISMA Statement [41].

Following the above steps, the obtained results are shown in Figure 2 which is an adaptation of the PRISMA flow diagram [41] and maps out the number of records identified, included and excluded, and the final obtained papers.

The different steps and outputs are summarized in Table 4.

### 3.7. Data Extraction

For each research question, a qualitative data analysis approach was followed to extract relevant data from the selected studies. The conducted process followed three major stages:

1. Papers were first read in detail localizing chunks of text related to each research question and were highlighted for further analysis. 2. Pattern codes and labels to assign symbolic meaning to the highlighted information were created. Related patterns were grouped in order to quickly find, extract, and categorize the segments relating to a particular research question. 3. A second in depth read of the text was performed and data was retrieved and stored in a spreadsheet (http://bit.ly/2Pjo7bQ) following the coding created during stage 2.

As a result, for each research question the following data extraction groups and labels were considered:

- Ecosystem goal: (IL) Independent Living; (OL) Occupation in Life; (R) Recreation; (HC) Health and Care; (O) Other
- Care and assistance services provided: (R) Reminder and information services (medication reminders, social events notification, alarm alerts); (S) Social interaction and support (social networks, teleconference with relatives/friends); (HC) Health or Care services (patient’s health physical or remote monitoring and feedback); (EM) Environmental Monitoring (alarms based on home sensors, errand detection) (RH) Rehabilitation and maintenance services (serious games, physical exercises); (O) Other. In those cases where a described top-level service involved two or more of these categories (for example, a doctor can elicit a medication reminder based on the patient’s health parameters) both service labels were considered.
- Who are the patient end users: (E) Elder users; (D) Disabled users, (CD) Patients with Chronic Diseases; (O) Others.
- Which actors that contribute to value co-creation are pointed out in the ecosystem: (HC) Health Care related actors (doctors, nurses, etc. either individuals or organizations); (P) Patients (patients and their next-of-kin); (F) Funders (Public funding agencies, private investors, etc.); (R) Regulatory parties; (SP) Software/technology Providers (provide software support or services, sensor devices, etc.) (GP) Goods Providers (pharmaceuticals or others); (ED) Educational / research institutions.
- How can actors add/modify ecosystem functionalities: (ID) Including new Devices; (ISO) Including new SOftware (MSO) Modifying existing Software; (NS) adding New Services; (MS) Modifying existing Services (e.g., doctors or authorities can change existing service contents.
- Ecosystem maturity: (C) conceptualization (e.g., an architecture and services proposal); (P) Piloted without real users; (PU) Piloted with real Users; (D) Deployed in real world
- Type of ecosystem architectures: We use the classification proposed by [42]: (C) Cornerstone Ecosystems; (S) Standard-based Ecosystems; (P) Protocol-based Ecosystems; (I) Infrastructure-based Ecosystems
- Software structure model notation and guidelines. Here we use the same notation as the one employed in [13]: (A) Ad hoc; (T) Tabular; (M) Metamodel; (C) Class diagram; (CM) Conceptual Map; (S) SNA.
- Sensor technologies employed for providing services: (A) Ambient sensors (e.g., temperature, humidity); (L) Localization and presence sensors (e.g., GPS, sonars); (B) Body sensors/medical devices (e.g., blood sensors, temperature sensors, EMGs, ECGs); (M) smart/Mobile technologies (mobile phones, PDAs, tablets, smartwatch, SmartTVs); (C) Cameras; (O) Other
- Sensors integration approaches: (N) not described; (P) Point-to-point; (M) Middleware based; (SO) Service Oriented;
- Do these technologies follow standards or are they ad hoc solutions: (S) Standards; (A) Ad hoc solutions
- Complementary training actions: (SD) on Software Development of new functionalities; (SI) on Systems Integration (e.g., adding new devices to the system); (TU) on Technology Usage (e.g., providing information to end-users/caregivers; (HE) on Health Education (e.g., best practices during rehabilitation); (O) Other

## 4. Results

In this section we present the results for each of the mapping and research subquestions obtained from analyzing the papers that resulted from the systematic process described in Section 3.6, for each of the research and mapping questions and after applying the data extraction process described in Section 3.7. Annotated results from the data extraction can be consulted at http://bit.ly/2Pjo7bQ:

### 4.1. Definitions of Technological Care and Assistance Ecosystems Given in the Literature—MQ1.1

In terms of the definitions given in the literature for technological ecosystems in health and care, nine studies were found which try to provide a definition at least related to the concept. The proposed definitions are summarized in Table 5. In [43] they describe a cooperative Health-IoT ecosystem by terms of the mobile Internet and health care value chains. In [44] they describe an application ecosystem for providing health and care services. In [45] they describe an IoT-based ecosystem and relate it with the definition of patient centered care (PCC). In [46] they provide a definition of a “Medical ecosystem” which includes physicians, patients and organizations. In [47] they provide a service oriented approach to the health ecosystem, where the definition is based on the service-dominant logic (SDL). In [21] they start from their previous definition of software ecosystem. After that they provide a description of the health ecosystem based on their architecture organized in three levels: software structure, business structure, organizational structure. In [48] they propose a definition based on merging different research and commercial ecosystem proposals. Finally, two more papers [10,49] describe the concept of ecosystem in terms of virtual communities that support processes in the elderly care sector.

### 4.2. Evolution Over Time of the Research Field of Care and Assistance Ecosystems—MQ1.2

**Figure 3:**
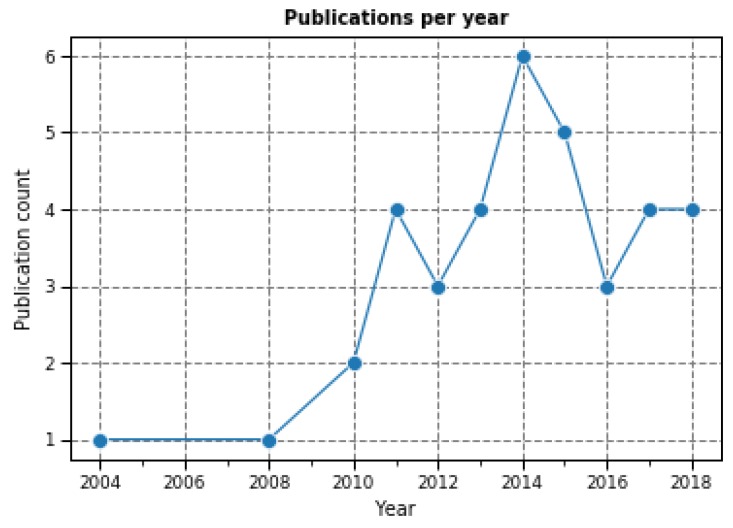
Number of publications per year in the considered studies.

Regarding the temporal distribution of works dealing with technological ecosystems in care and assistance, the evolution of selected works over time is shown in Figure 3. The only papers found before 2010 are [10,50].

It can be observed from the evolution of the number of publications that the topic of technological ecosystems focused in providing care and assistance services for people with special care needs is indeed an emerging field, as the only papers found before 2010 are [10,50]. It has to be noted that [10], which dates from 2004, refers to the concept of technological ecosystem as a virtual community of users. However, its implementation, and the actors’ interaction described in their work correspond to the ecosystem scope, so it could be considered as the first technological care ecosystem found in the literature.

### 4.3. Main Publication Venues—MQ1.3

**Figure 4:**
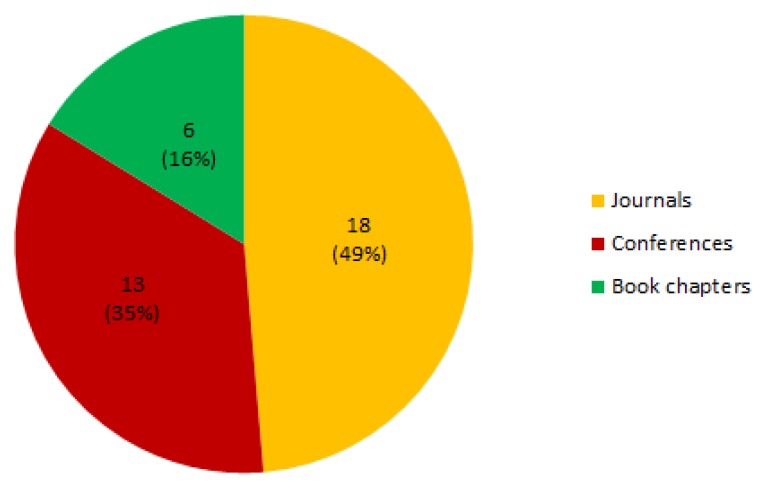
Publication types.

Figure 4 shows the distribution of the different publication types found in the reviewed papers. It can be seen that most papers are published in journals 18 papers (49%) or conferences 13 papers (35%), whereas six papers that passed our inclusion criteria have been published as book chapters. The publication sources of our papers are shown in Table 6. As expected from the results obtained during the search stage, the publication venues belong to very different research fields. This is due to the fact that, as identified in our PICOC model in Section 3.1 and ratified during the search process in Section 3.3, the population of our study is compounded by two unrelated populations: technological care and assistance ecosystems. In addition, it can be observed that the number of publications in journals is higher than those published in conferences. This contrasts with other areas such as Computer Science, where as stated in [51] “the number of conferences is significantly higher compared with the number of journals, which explains why most papers were published through this channel”.

### 4.4. Most Active Authors in the Area—MQ1.4

To answer this question we identified the authors from the selected papers. Only one researcher appears more than one time in the results, one as main author and the other as co-author of the paper. We have to note that this author also appeared in other excluded papers during our mapping process (Figure 2) due to the fact that they were studifications of the same implementation which described different components of the same ecosystem at different stages of development. Thus, the 37 papers included 167 different authors. The full list of authors is shown in Table 7.

### 4.5. Ecosystem Goal—MQ2.1

Regarding the ecosystem goal, we tried to evaluate how the different proposals approached the objective of providing care and assistance services to the end users. As such, results were grouped in terms of the four goals identified by [74] as the main groups of approaches for using technology in helping senior citizens or users with special needs:

- Independent living—assist in normal daily life activities e.g., tasks at home, mobility, safety, agenda management (memory help), etc.
- Health and care—health monitoring, disease prevention, and compensation for disabilities.
- Occupation in life—the continuation of professional activities along the aging process.
- Recreation—facilitate socialization and participation in leisure activities.

**Figure 5:**
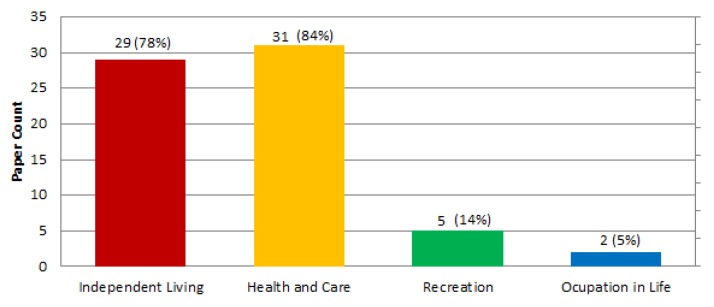
Number of papers per ecosystem goal.

Figure 5 shows the number of papers that refer to the different ecosystem goals. It can be seen that most proposals are focused either to health and care related services (31 papers, 84%), or to facilitate independent living (29 papers, 78%). On the other hand, only five proposals (14%) referred to recreation as one of their main goals, and just two proposals (5%) were focused on facilitating occupation in life to end users. Twenty-six (70%) papers addressed more than one ecosystem goal, and four papers [44,49,56,65] referred to three different goals in the ecosystem. When having more than one goal, almost all proposals combine independent living objectives with health and care related provision, except for [47] which combines recreation and health care provision, and [10,57] which combine independent living and recreation.

### 4.6. Care and Assistance Services Provided—MQ2.2

**Figure 6:**
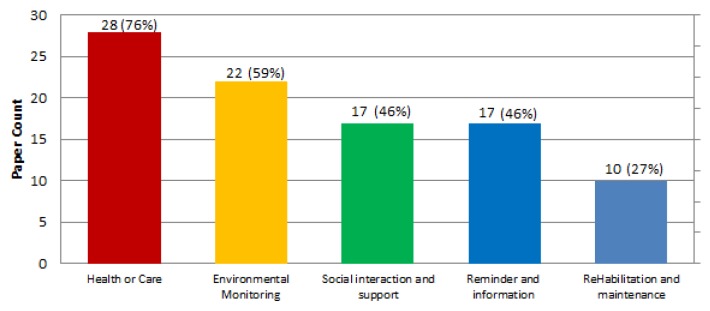
Number of papers per group of ecosystem services.

In terms of the care and assistance services provided, Figure 6 shows the different services provided in the studied ecosystems. The classification has been made in terms of encapsulating different services provided by the same entity (e.g., at a low level a device used in AAL could be used to provide different software services, at a high level a service provided by a care institution could be encapsulated as a single service if all services provided by that institution are related to the same objective).

Provided services are distributed in the papers as: health or care related services 28 papers (75%), environmental monitoring 22 papers (59%), reminder and information services 17 papers (46%), social interaction and support 17 papers (46%), rehabilitation and maintenance services 10 papers (27%). Examples of the different services are shown in Table 8.

### 4.7. Who Are the Ecosystem Actors—MQ2.3

**Figure 7:**
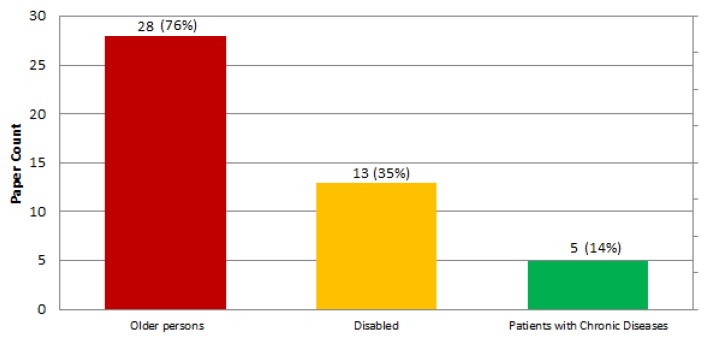
Number of papers per patient end user group.

We have identified three main groups of patient end users: older people, users with any type of disability, and patients with chronic diseases. Results show that older users are mentioned in 76% of the studies (28 papers), disabled users are referred in 35% of the studies (13 papers) and patients with chronic diseases are identified in 14% (5 papers). Figure 7 shows the results obtained for this classification.

**Figure 8:**
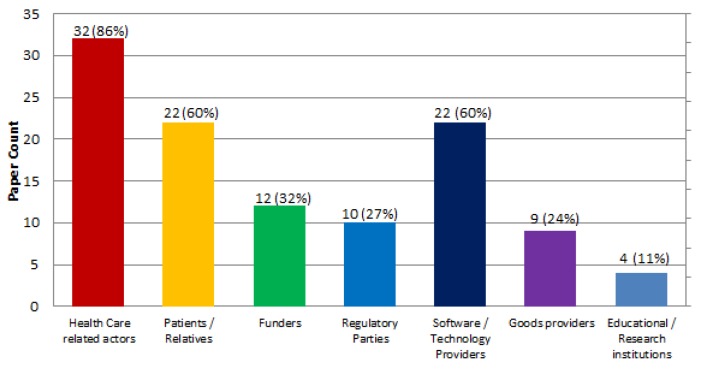
Number of papers per ecosystem actors.

In terms of the rest of the actors involved in value co-creation within the ecosystems, results are shown in Figure 8. We distinguish between: health care related actors, 32 papers (86%) (e.g., [6,7]); patients and/or relatives, 22 papers (60%) (e.g., [8,10]); funders, 12 papers (32%) (e.g., [54,58]); regulatory parties, 10 papers (27%) (e.g., [58,60]); software or technology providers, 22 papers (60%) (e.g., [62,63]); goods providers, nine papers (24%) (e.g., [43,64]); and educational or research institutions, four papers (11%) (e.g., [61,67]).

Regarding the specific value propositions identified in the papers for the different actors, we refer the reader to http://bit.ly/2Pjo7bQ for further information. It has to be noted that, in this case, patients are included in the patient and/or relatives group if they make use of the ecosystem to actively provide benefits in the value co-creation chain (e.g., doctors could use the data uploaded by the patients for creating tools that save costs through process enhancements).

### 4.8. How Can Actors Add/Modify Ecosystem Functionalities—MQ2.4

**Figure 9:**
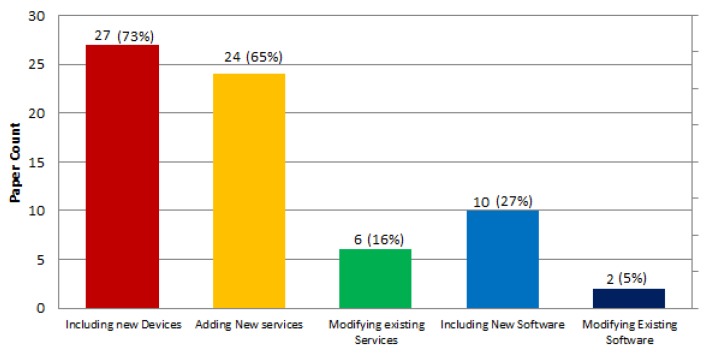
Number of papers per method for enhancing ecosystem functionality.

Figure 9 shows the results obtained regarding the different approaches allowed by the ecosystem proposals to enhance their functionalities. In order to analyze the results, we have considered only those enhancements that are denoted in the papers as possible means of improving the ecosystem functionalities, either directly or by means of the actors’ value propositions described in the text. According to our review, this enhancement can be achieved by including new devices in 73% of the cases (27 papers, e.g., [6,7,10,21]); adding new services, 65% (24 papers, e.g., [44,48,53,54]); including new software, 27% (10 papers, e.g., [47,58,59,64]); modifying existing Software 5% (two papers, e.g., [21,54]); and modifying or customizing existing services 16% (six papers, e.g., [55,61,66,70]).

### 4.9. Ecosystem Maturity—MQ2.5

**Figure 10:**
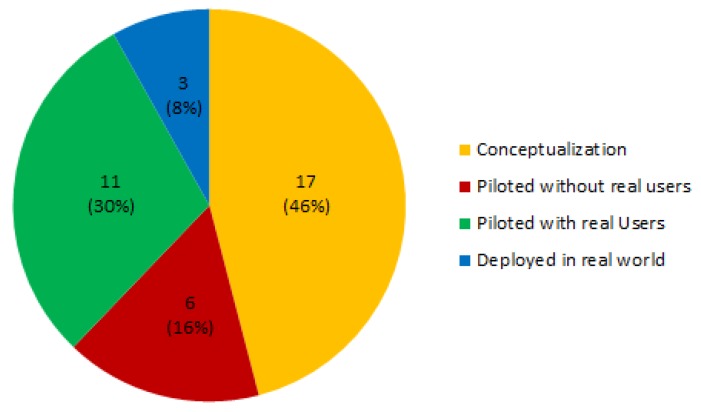
Distribution of papers in terms of the ecosystem maturity.

Figure 10 shows the results referring to the maturity of the proposed ecosystem solutions in the selected studies, obtained results show that 46% (17 papers, e.g., [9,47,48,53,63,64], etc.) describe an ecosystem conceptualization or have not been tested, 11% (six papers, [6,7,8,61,62,70]) have been tested either as a whole or any of their components have been piloted in lab conditions, 30% (11 papers, [44,45,56,66,69,72], etc.) have been piloted with real users, and finally only three papers ([11,60,67]) describe ecosystems which have been deployed in the real world.

The obtained results shown in Figure 10 are in line with the evolution of publications as observed in the Figure 3. As these kinds of technological ecosystems are an emerging field, papers describing ecosystem architectural proposals which have not been tested involve almost 50% of the studied papers. Papers referred as tested in lab conditions usually just test the software/technological structure of the ecosystem, performing simulations over a developed software architecture or implementing a particular use case (such as the provision of a specific care service). Those referred as having been piloted with real users imply proposals that have been tested in controlled environments, and usually with a relatively small and established group of ecosystem actors. These types of proposals are an implementation of the ecosystem but with reduced and controlled functionalities, and generally the tests focus on surveys and satisfaction questionnaires given to the ecosystem users.

Table 9 sums up the results found in terms of the concrete methods employed for testing the different proposals. They include using questionnaires to evaluate the stakeholders (patients, carers, service providers, etc.) satisfaction or the ecosystem usability, evaluations on the performance of the whole running ecosystem (e.g., system logs on service demand or number of users, memory load, network status), evaluations on some components (e.g., middleware for connecting sensors, technologies for providing services) and simulation based evaluations. Finally, it has to be noted that some ecosystem conceptualizations included meetings with stakeholders or surveys focused on stakeholder preferences during their design process, either for obtaining ideas on their preferences or for obtaining assessment in proposed ecosystem use cases.

### 4.10. Ecosystem Architectures Proposed in Care and Assistance Environments—MQ3.1

Regarding the ecosystem architectures proposed in the papers, we follow the ecosystem architecture classification proposal described in [42], and distinguish between four types of ecosystems:

- Cornerstone Ecosystems: where actors interact on top of a common software platform and usually extend the platform’s functionality. Thus the existence of a technological platform is of central importance for an ecosystem of this type. The structure and governance of this type of ecosystem is usually centralized. Examples of this type of ecosystem are Android or the AppStore.
- Standard-based Ecosystems: where instead of a common platform, the compliance to standards is the key requirement for contributing in the ecosystem. Usually, compliance to standards is set above the functionalities and concrete realization of the contributions.
- Protocol-based Ecosystems: where a protocol API is shared among all actors, providing more flexibility over technical contributions to the ecosystem. Protocols are a less restrictive and more flexible technical linchpin of ecosystems. They provide a predefined specification of interaction of contributions with each other (e.g., exchange of data, call of software services).
- Infrastructure-based Ecosystems: where a common technology is shared among all actors, providing tools at development time but at the same time maintaining independence on the contributions. Usually, the interaction among actors are on a social level. Examples of this type of ecosystem are Gnome or Github.

**Figure 11:**
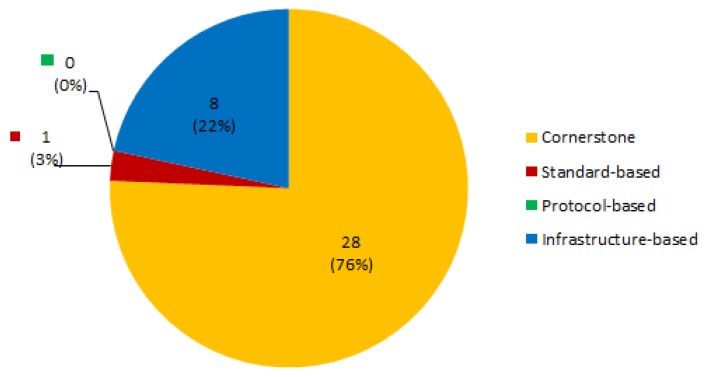
Percentage of papers per ecosystem type.

Figure 11 shows the distribution of papers in terms of the typification of their architecture as described above. It can be seen that the great majority of the proposed architectures follow a cornerstone approach (28 papers, 76% of the total), describing a central technological platform over which actors develop their activities (e.g., [55,56,57,59,69,70,71,72]). Then eight papers (21%) can be typified as infrastructure-based ([8,9,11,46,48,60,61]), and one paper (3%) [58] describes a standard-based ecosystem. However, it should be noted that [58] could also be considered as a protocol-based ecosystem, as their objective is to provide a specification for health and care technologies communication, but from a standardization perspective. Although many papers describe to some extent the ontologies and protocols of their proposals, no papers were found whose main objective was providing a protocol API for care services provision without referring a central platform.

### 4.11. Model Notation and Guidelines for Describing the Ecosystem Architecture—MQ3.2

To answer this question, we followed a similar approach for classifying the different approaches as the one described in [13] for open source software ecosystems. By using a similar approach, we are able to compare if the modelling techniques employed in other more consolidated technological ecosystem domains (such as software ecosystems) are also applied for modelling care and assistance ecosystems.

As such, in [13] differentiates the following representations in order to categorize the modeling techniques employed in describing or visualizing the ecosystem: Ad hoc representations, tabular, metamodels, class diagrams, conceptual maps and social network analysis (SNA) representations. It has to be noted that we selected the dominant modeling technique in the case that more than one type of representation is used.

**Figure 12:**
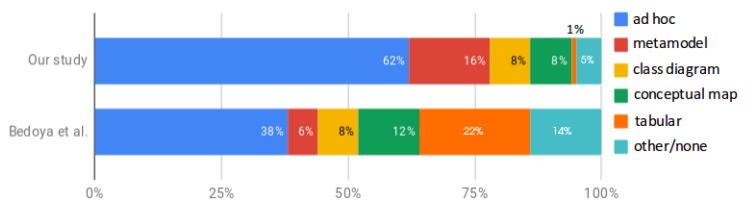
Comparison of percentage of papers in terms of modeling notations.

Different modeling techniques were found, being the ad hoc notation the predominant one as present in 23 papers (62%, e.g., [6,7,8,9,11]), followed by meta-modeling techniques in six papers (16%, [21,48,54,59,68,71]), class diagrams in three papers (8%, [10,43,70]), conceptual maps in three papers (8%, [67,72,73]) and tabular representations in one paper (3%, [45]). Only one paper did not provide any kind of representation [47]. Figure 12 shows the different modeling techniques found, along with the numbers obtained by [13] for the case of open source software ecosystems.

### 4.12. Sensor Technologies Employed for Providing Care and Assistance Services—MQ3.3

**Figure 13:**
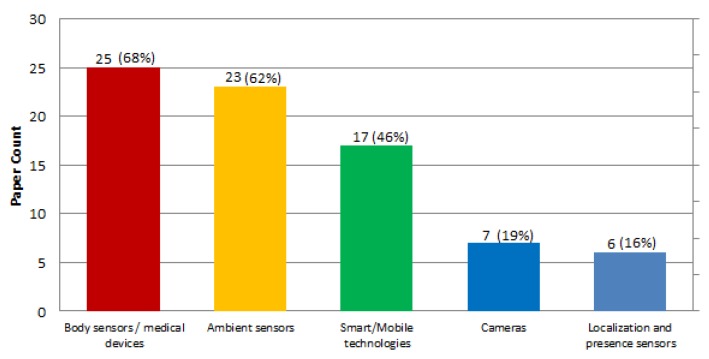
Number of papers per technologies employed for providing services.

The objective of this question is to explore which are the main types of devices mentioned in the literature as to provide health and care services to the objective end users. We have grouped the technologies that compound the technological infrastructure of the ecosystems mentioned in the reviewed papers into five categories: ambient sensors, localization and presence sensors, body sensors or medical devices, smart or mobile technologies and cameras. Figure 13 shows the distribution of the different technologies among the papers. It can be observed that the most commonly considered technologies are body sensors and medical devices cited in 25 papers (68%), followed by ambient sensors cited in 23 papers (62%), smart technologies mentioned in 17 papers (46%), cameras mentioned in seven papers (19%) and localization and presence sensors in six papers (16%). It has to be noted, though, that many papers just provided a general citation of the type of sensor to be included (e.g., “medical sensors”), or stated that their proposals are open to different type of devices without naming the type of device to be included.

Examples of the different technologies described in the studies include:

- Body sensors and medical devices: ECG devices [46,47,63]; blood analyzers [62,66,68]; body thermometers [62]; etc.
- Ambient sensors: temperature [6,7,66]; humidity [63,65]; gas detectors [65,66]; etc.
- Smart technologies: smartTV [8,53] PDAs [53]; mobile phones [48,71]; smart gloves [45]; etc.
- Cameras: [52,57,67,72]
- Localization and presence sensors: infrareds [7]; RFID [7]

### 4.13. Sensor Integration in the Ecosystem—MQ3.4

In terms of the integration of sensor devices in the ecosystem architecture, it is of particular interest to investigate the approaches followed to obtain and manage the information gathered from the different devices. The current trend in IT solution providers for health (including the care and assistance environment) is to move from fragmented services to progressively more integrated ones, which are likely to be provided by multiple stakeholders through well-elaborated collaboration mechanisms [3]. Thus, providing care services requires the utilization of a large number of sensors and devices, that are either developed ad hoc or obtained commercially off the shelf, and which need to be integrated with the other parts of the ecosystem [55].

**Figure 14:**
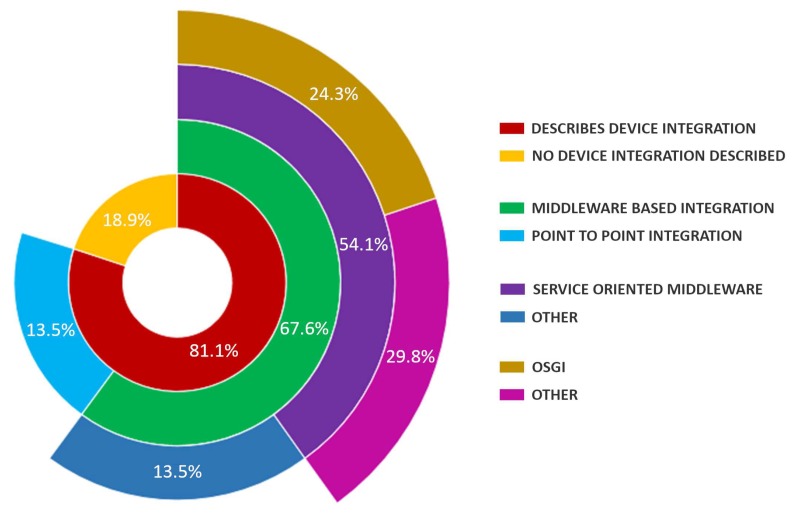
Percentage of papers for the different integration approaches.

Figure 14 shows the distribution of papers in terms of the integration of sensor technologies and devices described in the studied ecosystem proposals. It can be seen that, of the papers that describe how integration is (or will be) carried out (81.1%), a high percentage choose a middleware based integration (67.6%) over the conventional point to point integration (13.5%). Point to point integration approaches found in the literature rely on a gateway (custom made [43,63], tablet [8] or set-top box (STB) [44,64]), on which specific software is developed in order to gather, aggregate and transfer data from the devices to the upper ecosystem infrastructure using conventional protocols (Zigbee, Bluetooh, RFID, etc.).

On the other hand, middleware based approaches mainly follow a service oriented architecture as 80% of those papers refer to a service oriented middleware (SOM) (54.1% of the total number of papers). Other middleware approaches include agent-based [10], fog nodes [45] and database based solutions [46,67]. SOM integration described in the papers encompasses different techniques such as web services [60,66], microservices [6], services over a device abstraction using xml-based ontologies [65], services over cloud based infrastructures [68,70]. It is also noticeable that almost 50% of the papers that follow a SOM approach make use of the Open Services Gateway Initiative (OSGi) framework [52,53,54,55] (24.3% of the total number of papers).

### 4.14. How Sensor Technologies Take into Account the Care and Assistance Environment—MQ3.5

Technologies involved in care or assistance solutions require a degree of regulation to ensure that they are safe, and that the data is shared and processed in a uniform, consistent and safe manner. The degree of regulation required depends on the level of risk associated with the technology. Although the list of regulations is broad, and in many cases they are geographical and domain-specific [75], with this question we wanted to explore the different regulations and standards referenced in the different ecosystem proposals. Following health standards is not an easy task and usually many solutions do not take them into account during the initial steps of development. However, when used in digital health solutions these standards provide many benefits as they allow the exchange of information in a safe and effective way, facilitate clinical decision support and enable data to be aggregated, leading to better outcomes. Overall, the found cited health-related standards include:

For medical records:

- Health Level-7 or HL7 [76] refers to a set of international standards produced by the Health Level Seven International and adopted by the American National Standards Institute and the International Organization for Standardization. They provide standard guidelines for transfer of clinical and administrative data between software applications.
- XDS.b [77] is an standard promoted by the Integrating the Healthcare Enterprise (IHE) for exchanging a variety of different document types across a network of independent healthcare providers.

For sensors or medical devices:

- CEN ISO/IEEE 11073 [78]—to enable communication between medical, health care and wellness devices and with external computer systems. Provides means to communicate devices with upper levels (for example with HL7)

Other:

- ISO 13940:2015 [79]—covers the concept definitions needed whenever structured information in healthcare is specified as a requirement. The definitions are intended to refer to the conceptual level only and not to details of implementation.
- HIPAA [80] (Health Insurance Portability and Accountability Act) (software)—USA law approved in 1996 which establishes (among others) standards for electronic health medical transactions

Special mention needs to be given to [54] where some of the architectural models were intended to become an standard, as part of strategic groups [81] or to the standardization of use cases related to AAL [82] among others. In addition, in [58], which is a standard- based ecosystem where they propose their own standards for both medical records transfer and sensors or medical devices communication.

**Figure 15:**
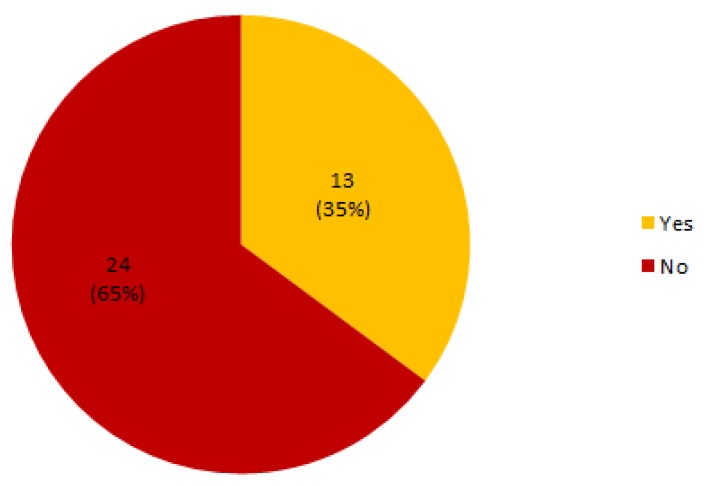
Percentage of papers that consider standards in their proposals.

Table 10 resumes the papers that considered medical standards in their solutions. Although many of them refer to different types of standards in their solutions such as Zigbee, Bluetooth or KNX, it can be seen that just 13 out of 37 papers (35%) take medical standards into account (see Figure 15). We refer the reader to http://bit.ly/2Pjo7bQ for further information about the standards considered in each paper.

### 4.15. Complementary Training Actions—MQ3.6

Keeping up with technology in terms of adopting and ultimately employing a new technology is a difficult task, but it could become even more difficult for senior or disabled persons who may present different impairments in terms of their sensory abilities. For that reason, training actions on technology usage is important in ecosystems related to technological solutions for care and assistance. In addition, in terms of adding new value to the ecosystem and enhancing the ecosystem functionalities, software and device providers may need reference documents and sites where they can obtain information on how to adapt their devices to the ecosystem ontologies, or how to develop new software that provides new services or enhances the existing ones. For that reason, this question aimed to investigate the different training actions proposed in the studies.

**Figure 16:**
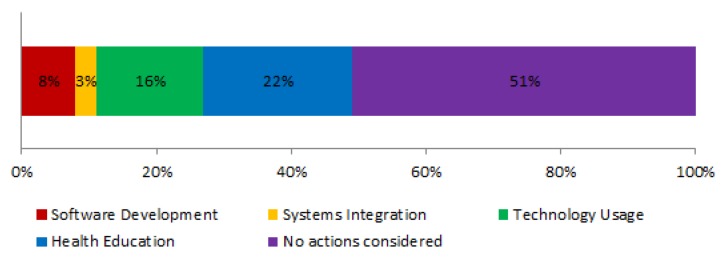
Percentage of papers per training actions.

Figure 16 graphically shows the different proposed training actions. Overall, only 10 papers considered them in any form, and they can be grouped as: on health education eight papers (22%); on technology usage six papers (16%); on Software Development of new functionalities three papers (8%); on systems integration one paper (3%). Table 11 gives a brief description of the different training actions considered in the papers.

## 5. Discussion

In this section we discuss the findings related to our main research questions described in Section 3.1 based on the results obtained for the different mapping questions which can be consulted in (http://bit.ly/2Pjo7bQ) and which have been surveyed in Section 4.

### 5.1. How Are Care and Assistance Technological Ecosystems Approached in the Literature—RQ1

#### 5.1.1. Ecosystem Definitions

In order to explore how technological ecosystems for care and assistance are considered in the literature, we must first consider what a care and assistance technological ecosystem is in terms of its definition. As stated in [48], “there is not a single concrete definition that encompasses all the characteristics of a health ecosystem”, a statement that is reflected in the results shown in Table 5, which presents different types of definitions for care and assistance technological ecosystems from different perspectives (see Section 4.1). In this sense, the work of [20] focused on defining digital health innovation ecosystems, by analysing 65 studies and combining the main concepts found for three different themes: definition of digital health, definition of innovation, definition of digital ecosystems. Thus, their definition of digital health innovation ecosystems resulted in: “a network of digital health communities consisting of interconnected, interrelated and interdependent digital health species, including healthcare stakeholders, healthcare institutions and digital healthcare devices situated in a digital health environment, who adopt the best-demonstrated practices that have been proven to be successful, and implementation of those practices through the use of information and communication technologies to monitor and improve the wellbeing and health of patients, to empower patients in the management of their health and that of their families”.

Considering this definition of digital health ecosystems, which includes those devoted to care and assistance services but also other health related areas, we can follow a similar approach as the one described in [20], and look for occurrences in our revised literature of the three main concepts involved in our ecosystem: care and assistance, technology and ecosystems. Results shown in Table 12 denote that in fact, although definitions may vary from the point of view of the technologies and ecosystem goal, most definitions share common characteristics in terms of providing technological means to a community of users which consist in patients, relatives, carers, doctors and organizations in order to involve all of them in providing better care and assistance related services.

#### 5.1.2. Evolution of Publications

Research in new technologies for care and assistance is a growing research area and the domain has evolved at a fast pace during the last two decades in various directions [4]. This tendency is also reflected in terms of the adoption of the concept of software and technological ecosystems and the development of new solutions related to this type of solution, which has increased from 2003 onwards with special emphasis during the last decade as shown in other systematic reviews [12,13]. In terms of the evolution of the reviewed publications over the years, this trend can be observed in Figure 4, where most papers where published from 2011 onwards (78%). In addition, considering that 2007 was the start of different European research programs focused on care and assistance services such as the AAL Joint Association (AAL EUROPE 2007) [4], this discrepancy between before and after 2011 can be considered reasonable. In fact, many papers included in the review such as [52,57,59,64,68,70,71] correspond to European funded projects.

In terms of the scope of the publications, until 2011 the topic of the research was clearly focused on developing software platforms for providing integrated care services [10,49,50,53,62,73], but later other approaches were taken into account such as mobile social networks [11,49], or cloud based solutions [8,46,61]. The decreasing trend observed in the last couple of years is possibly due to the delay in the indexing performed by the selected sources.

Apart from analyzing how care and assistance technological ecosystems are approached in the reviewed literature, the purpose of our second and third research questions is to have a notion of the main components that compound this type of ecosystem and how the reviewed papers approach them. The main structures that are comprised by a technological ecosystem have been proposed by Christensen [21]: software structure or technological structure, the structure of software elements and devices that form the core of an ecosystem; the business structure, how ecosystem actors create value (in a for-profit or non profit manner) and the organizational structure, how the interaction and organization of actors and software are governed, which is in turn related and dependant on the ecosystem architecture (e.g., for an actor to provide care services in the ecosystem). The next sections discuss these aspects from the results obtained in Section 4.

### 5.2. Main Distinctive Characteristics of Health and Care Ecosystems—RQ2

#### 5.2.1. Ecosystem Goal and Services

In the care and assistance domain, earlier proposals focused on techno-centric solutions that aimed to provide specific healthcare related services to senior citizens or disabled persons [83]. However, more recent proposals such as the ones reviewed in this paper extend this concept to “active ageing” or “productive ageing”, modifying the perspective of the society when considering the ageing process [5]. As stated in [3], the effective support to the ageing process should not be exclusively focused on providing healthcare to senior persons, but also on the notion of “productive ageing”, which involves living as independently as possible, having an occupation, and being able to maintain a correct physical and mental status on their own. These considerations are reflected in the reviewed studies, as most ecosystem proposals consider the provision of tele-health related services as one of their goals (84%), but combine them with other types of main goals described in Section 4.5 [44,56,59,64,65,68,69,70]. In this sense, however, apart from healthcare provision, reviewed papers are mainly focused on independent living of the senior or disabled users and on providing them with means of independent living. A lack of focus on the goals of occupation in life and recreation can be observed in the reviewed papers as only five proposals (14%) referred to recreation and just two proposals (5%) were focused on facilitating occupation in life to end users (Figure 5).

Obtained results are correlated with other systematic reviews such as [84], focused on trends and user perspectives in devices for ambient assisted living. They analysed 48 studies in the field and concluded that the majority of technologies are mainly focused on gathering, processing, storing and transmitting the data obtained in order to monitor the health and wellbeing of the dependant users.

As defined in [85]: “A care and assistance service may involve a number of software services and human intervention. The actual structure of such a service also depends on the interaction between the provider and the user, and may ultimately (and dynamically) vary according to the flow of that interaction.” For that reason these services may involve atomic software and technological services along with human intervention. In this respect, most reviewed papers provide a wide range of combined services in order to achieve the main ecosystem goal, a trend that has been already reported in other literature reviews related to technologies for care and assistance provision [4,84,86].

For example, in terms of a health and care focused ecosystem, in [60] they depict the following services: remote physiological data transmission and management, health records transmission and management, health and care education services (health education, drug use) and remote health education learning. In terms of an independent living focused ecosystem, in [52] they consider: personal activity and household assistant, monitoring of preventive and rehabilitation sports, sensor-based activity, management of the patients’ health data, sensor-based fall prevention and fall recognition, alert communication, consult personal health record. In [44], which is also focused on recreation, they describe: cognitive Stimulation, physical exercises, socialization (Facebook) (Games); News. It can be observed from the obtained results (and also in related recent literature [5,87]) that the current trend in care and assistance ecosystems is to evolve from fragmented isolated services to progressively more integrated ones which could be deployed over a common infrastructure.

#### 5.2.2. Main Actors and Value Propositions

As shown in Figure 8 in terms of the actors considered in value co-creation in the ecosystems, they mainly include health care related actors (86%), patients and/or relatives (60%) and software and technology providers (60%).

In contrast with this finding, in the systematic review performed in [4] they classify the stakeholders that appear in the AAL solutions into four categories: physicians, caregivers, relatives and patients. Their results show that patients as end-users appear in 100% of the studies considered, followed by Physicians and Caregivers and Relatives, while the combination of these categories patients + caregivers + relatives, patients + physicians + caregivers, etc.) only appear in 51% of the 236 studied proposals.

However, as discussed in Section 4.7, our review focuses the search on the technological ecosystem concept, beyond specific solutions for AAL, infrastructures for AAL or just systems of systems. As such, their results do not reflect the concept of technological ecosystem that must include evolutionary and scalability capabilities and describe the added value that the actors provide to the ecosystem from a business, organizational or technological perspective.

Value co-creation is a fundamental aspect in the development of ecosystems of any nature which has gained importance through the years. This tendency is shown in [88] where they studied value co-creation in 421 studies ranging from 200 to 2012, and found that more than 70% of the reviewed papers dated from 2010 onwards.

As stated in [47]: “the motivation of actors to participate and engage within a medical ecosystem arises from the reciprocal benefits, namely the value propositions that variant types of actors within the ecosystem offer and seek”. As such, value co-creation in ecosystems emerges through interactions and collaboration between the different ecosystem actors [89], so it can be employed as a good indicative of the community of actors that take part in the ecosystem.

However, as the goal of the searched technological ecosystems is to provide services to people with special care needs, it is evident that the value offered by these ecosystem actors will always be present in the interaction with other actors as found in [4], so we have made a distinction in terms of their contribution as “active” and “passive” end users. As such, “passive” patients are the ones who are the main target of the ecosystem but do not provide any added value apart from being potential “care and assistance service customers”. In most of the reviewed papers, the group of end users is highly correlated with the ecosystem goal and provided/proposed services (i.e., if patients with chronic diseases are considered, mainly healthcare oriented services are proposed [47,48]). As stated in [90], actors as a foundation resource define much of the structure of a service ecosystem by creating locally stable conditions for value creation, and service ecosystem processes.

However, in spite of the observed tendency towards broadening the scope of the ecosystems [5,91], this can be an indicative that, from a business perspective most of the ecosystems proposed in the reviewed literature follow a customer-centric approach rather than a stakeholder-centric approach. The early stages of development of the solutions found in the reviewed papers (Figure 10) suggest that they still have not evolved from a end user-centric perspective.

During the first steps of technological ecosystem design and planning, it is important to take into account which are the potential ecosystem actors, along with which could be their main interests in taking part in the ecosystem community. The main problem is not considering which values or benefits are sought or offered to the ecosystem community by the ecosystem actors by means, for example, of performing a previous exploratory study. For example, in terms of value co-creation, the core value sought by medical and technological companies from a care and assistance ecosystem is business growth. Another example could be non-profit organizations that already provide certain care services offered by the ecosystem but may be reluctant to incorporate certain technologies to provide those services if the benefits of doing so are not clearly stated. Therefore, even if a medical application or device could improve individuals’ lives significantly, if the value proposition is not mutually beneficial it might not be feasible to develop or include into the ecosystem due to a lack of interest of the stakeholders. This type of stakeholder exploratory study in the revised papers is only found in [21,47,54].

Not considering the actor’s value propositions during the ecosystem design stage is a flaw that has been reported in the literature [21,92,93]. In their extensive review on value propositions from an ecosystem perspective, in [92] they show that value propositions act as a balancing mechanism in the service ecosystem and if ecosystem actors do not perceive value propositions as mutually beneficial (i.e., they do not perceive value co-creation within the ecosystem), changes may occur in the ecosystem which involve: actors may change the value they offer to the ecosystem, they may try to develop new relationships in which value propositions are reciprocated, and finally the ecosystem may collapse if there is a great misalignment between value offered and value sought.

#### 5.2.3. Adding Functionalities to the Ecosystem

In terms of adding new functionalities to the ecosystem, the three main considered groups of actors found in the studies (care related actors, patients/relatives and software/technology providers) conform the main group of ecosystem stakeholders on which the development of new functionalities depends (see Section 5.2.2). As such, in terms of expanding the ecosystem with new functionalities, the three main methods considered in the papers are: including new devices in 73% of the cases, which mainly depends on health care related actors and technology providers, adding new services, which mainly depends on software providers and health care related actors, and including new software (27%), which mainly depends on software providers/developers (Figure 9).

Obtained results differ from other areas of ecosystem development, for instance in software ecosystems, where including new software is one of the main goals in ecosystem evolution and sustainability [13] or eLearning oriented ecosystems, where the evolution is mainly focused on including new learning content [94].

On the other hand, research institutions and regulatory parties are barely mentioned in the reviewed literature as ecosystem complementors who could include real enhancements to the ecosystem (27% and 10% respectively). This also happens in other systematic literature reviews regarding ecosystems in other fields such as [12,13,36,95]. However, there are fields where regulatory parties take a main role in the ecosystem evolution such as in the automotion [96]. As such, main methods for expanding ecosystem functionalities highly depend on the scope of the ecosystem field and related actors.

In the particular case of care and assistance ecosystems, the reviewed literature shows that the capacity of adding new functionalities to the ecosystem, that comes from health care related actors and patients or relatives, is mainly reduced to assessing or complementing the existing services, providing new service ideas or generating new necessities for the creation of new services or the modification of existing ones. Technology and software providers are the main actors in charge of implementing those necessities into real enhancements in the ecosystem, provided that the ecosystem is open to new functionalities. However, as stated in [97] regarding software systems, “open and closed systems have been viewed as opposites. We argue that they are not open or closed,… but there is a continuum between open and closed. Therefore we consider the dichotomy of open and closed as false, as there are more options. Open systems are popularized by the open-source community, but this can be misleading. The characterization source in “open-source” says it all; it is only about code in this case, not about other organizational processes or software parts. Open standards, open formats and open source are often considered equivalent.”

Although from the obtained results, the fact that many proposals (which mainly describe platform-centric or infrastructure-based solutions) state that they are open to include new technologies (e.g., by aggregating new medical devices [45]), does not necessarily imply that they are in fact open to offering new care services capable of attracting new stakeholders, developers and end users (which in turn are one of the main stimulus for developing open ecosystems [47]). For example, regarding physiological monitoring, adding a blood meter to a set of existing physiological sensors does not imply providing a new service, but rather enhancing the outputs of an existing one. Thus, the added value of this addition may be little from an ecosystem perspective.

On the other hand, in terms of the openness of the proposals to include new software enhancements, it has to be noted that as cornerstone architectures and infrastructure based ecosystems are the main types of ecosystems found, so the degree of ecosystem openness mainly depends on the development of new services over existing infrastructures, such as providing new applications to a mobile based care ecosystem. This is reflected in our results as only 5% of the reviewed papers provide means to modify the existing software.

This contrasts with purely software ecosystems, where the recent systematic review carried out in [13] found 87 relevant papers dedicated to open source software ecosystems. This is probably due to the different business model followed (user-oriented vs. technology-oriented), the evolution capacity of the care and assistance ecosystems which need developments sufficiently mature and tested to be incorporated, as well as to possible implications derived from openness in terms of data privacy. In any case, and as seen in examples such as [98,99,100], this trend is changing in terms of care and assistance oriented ecosystems.

#### 5.2.4. Ecosystem Maturity

Regarding this ecosystem maturity, Figure 10 shows that only 38% of the papers have been tested with real users, and that only three have been deployed in the real world. These papers include [11], which consists of a mobile social network platform for the communication between professional communities and families with children with developmental disabilities (CDD), Ref. [60] where they propose a closed telecare information platform based on service oriented architecture (SOA) for providing in-home health services, and the [67] which describes the Behavior Connect platform which can connect caregivers to their healthcare providers, and collect annotated video data from children with autism disorders for behaviour analysis. It can be seen that although deployed in the real world, these proposals are infrastructure based ecosystems which provide care and assistive services over an existing infrastructure, such as the mobile network, the existing hospital and health services and the Behavior Connect commercial platform.

These results are correlated with the ones obtained by [4], where they show that out of 236 studies that included technologies in the AAL domain, only 10 papers took into account all groups of AAL related stakeholders, which they attribute to the “technical or technological immaturity or lacks in AAL needs and requirements analysis.”

The lack of real world deployed ecosystems described in the reviewed papers may be due to the fact that many of them are recent ecosystem proposals; no additional scientific work has been done after the papers were published or efforts were redirected to the real ecosystem deployment. In this sense, a further online search was performed and we found that some of the conceptual proposals have in fact been partially or completely deployed such as the univerSAAL platform described in [54], which is an open platform now available at [98] and provides all the necessary components for development care solutions and device integration; it includes a wiki and documentation for developing care and assistance services over it. The openAAL open source middleware platform, which is based on the work developed in [54], but which provides additional functionalities, is available at [99]. There is also the OpenTele platform available at [100], which is related to the work presented in [21] and consists of a telemedical platform for handling data and measurements from personal health devices.

### 5.3. How Sensor Technologies Are Integrated in the Ecosystem Proposals—RQ3

#### 5.3.1. Ecosystem Architectures

First of all, it has to be noted that there is a lack of concrete classifications in the literature in terms of technological ecosystems’ architecture no matter what their scope is [12,13,34]. The closest work on technological ecosystems typification has been developed by Knodel [42], based on the study of [12] who identified that three main structures could be found in software ecosystems: common software or technological platform, business or interests, and connecting relationships between ecosystem actors. From there, in [42] they provide a proposal for software ecosystems classification based on these three main structures as described in Section 4.10. However, as they state, there is still a need for a well-defined taxonomy of technological ecosystems and their characteristics, because depending on its type, the ecosystems expose different characteristics in their structure, governance, and the adoption of changes. One of the main conclusions of their work is that there also exists a lack of common platforms devoted for technological ecosystems in different domains, such as software [13], gaming [47] or telemedicine [21].

In terms of care and assistance related solutions, the need for common software platforms has also been denoted. For example, in a recent survey of secondary studies on AAL platforms published between 2013 to 2017, they highlight the absence of a reference architecture that can be used to create a standard for AAL platforms [87].

Related to those conclusions, our results in Figure 11 show that most of the papers’ main efforts are focused on developing ecosystems where actors interact on top of a common technological platform (76%) where the structure and governance of this type of ecosystem is usually centralized. Regarding the proposed platforms that sustain this type of ecosystem architecture, different approaches are found in the reviewed papers, which range from service oriented architectures [9], agent based [10], layered [9] or based in web services [71]. In addition, due to the complexity of this type of solution, many combine different software architectures within the central platform.

On the other hand, as [85] affirms, most care and assistance developments found in the reviewed papers are usually too techno-centric (a trend also reported in [84,86]), so care services are too fragmented and provided by different unrelated stakeholders. In fact, it can be observed from our results that the other main ecosystem architecture type considered in the literature consists in ecosystems that share a common infrastructure, such as cloud [21,46,61] or mobile [49,73], where this common technology is shared among all actors and it provides tools for the development of new applications while at the same time maintaining independence on the contributions.

It has to be noted that although the ecosystem typification proposed by [42] is well described in terms of ecosystem governance, structure and actors’ relationship, it is a first classification approach focused towards describing software ecosystem. Due to the characteristics of technological ecosystems which, involve a broad range of technologies and fuzzy interactions, it is difficult to categorize the architectures found in the selected papers. For that reason, no studies have been classified as protocol-based ecosystems even though many of the papers describe topologies for device, software or even actors’ interaction. The fact that in these cases the governance of the ecosystem is centralized over a common ecosystem platform made us consider those proposals as cornerstone ecosystems. Finally, only one ecosystem proposal was found to be standard-based [58], which we consider due to the fact that scientific publications are not the common venue to publish this type of work.

This lack of standard-based ecosystems studies in the scientific literature can also be found in other application domains such as [12,36,95].

In terms of the modelling techniques employed in the literature, we observed in Section 4.11 that as happens in the case of other types of technological ecosystems, such as the one analysed in [13] (see Figure 12), most of the papers use ad hoc models to support their works or employ available modelling techniques. As argued by [13] the development of new modelling techniques is important in software ecosystems because they have evolved from different domains. This is even more pronounced in the case of care and assistance ecosystems, because as we described in Section 3.1 and has been confirmed through or review process, the areas of technological ecosystems and care are two very distinct population dimensions.

#### 5.3.2. Employed Sensor Technologies and Devices

In terms of the employed technologies, we observe a wide range of technologies which have been considered in the selected studies (see Section 4.12), and almost all papers cite the combination of one or more technology to be supported (e.g., [10,53,57,73]). This is expected as services provided in ecosystem environments usually involve many different stakeholders and objectives by definition; this is related to other surveys that explore the tools employed for assistance provision [84,86].

In addition, as reflected in the literature [84], employed technologies named in the reviewed studies are in correlation with the goal of the ecosystem, i.e., ecosystems aimed at enhancing independent living mainly focus on describing ambient and localization sensors [65,72], whereas those whose main focus is on health related services identify body sensors and medical devices in their solutions [48,60]. Platform based solutions are conceived to accept a wider range of different technologies [6,52,71].

It has to be taken into account, however, that many proposals are conceptual models (Figure 10) based on open platform proposals [54,57] and as such are designed and described to be open to including new devices. An interest in developing platforms or solutions open to the integration of devices of many different kinds has been observed, which may be explained by the lack of commercial state of the art care and assistance related platforms. In his work, [101] analyses different existing commercial AAL related platforms and concludes that all of them are proprietary and partially closed where only a limited list of third party sensors can be integrated. In this sense, many studies do not provide enough information on how these technologies are integrated with the rest of the ecosystem components, mainly those which are infrastructure-based which take this process for granted. In those that do, the inclusion of the care technologies in the ecosystem architecture is usually made by means of a middleware layer that can be either developed from scratch as part of the central platform proposal [66], or employ different existing middleware platforms such as the OSGi specification [7,52,54,72].

In terms of how these technologies take into account the care and assistance environment they are supposed to operate in, we have to note that only 35% of the papers take medical standards into account in some way or another (see Figure 15). The need for standardization has also been noted by other authors such as [102], who performed a survey focused on M2M Systems for mHealth and concluded that “apart from the specific challenges in each aspect of the mHealth systems, efforts must be concentrated on standardization activities, that will enable the market exploitation of the scientific contributions”.

It has to be considered that technologies that are intended to operate in close relation with humans, and particularly those involved in health, care or assistance solutions, require a degree of regulation to ensure that they are electrically, chemically, biologically, and physically safe for the patient, and that the data is shared and processed in a uniform, consistent and safe manner (for example in the case of medical records). We see a lack of concern in standard acceptance in the studied papers, which could be related to the ecosystem maturity of the proposed solutions. This issue has also been addressed in other studies related to devices in care technologies such as [103], where they review 25 papers related to applying wearable systems for monitoring mobility-related activity in individuals with chronic disease conditions, and conclude that “evidence-based clinical applications of these methods in individuals with chronic diseases are in need of further development”.

#### 5.3.3. Sensor Integration in the Ecosystem

In terms of the integration of sensor technologies into the ecosystem, results presented in Section 4.13 show that a great percentage of the studied papers follow a middleware based approach. This is probably because, in the case of care and assistance ecosystems where there is a great number of different components that interact with each other (see previous section), ad hoc integration turns out to be complex and even infeasible in some cases, as integrating such a large collection of devices, which employ different protocols and standards, can lead to a chaotic integration situation [55].

The constraints of the ad hoc integration are reflected in the studied papers that follow a point to point integration approach where, although a specific middleware is not used for the integration of devices, researchers are compelled to employ different kinds of dedicated gateways so the data obtained from the sensors are accessible by the rest of the ecosystem components to provide aggregated services. Although these gateways serve as an abstraction from the specific protocols and standards of the interconnected devices to the upper layers, data gathering and aggregation must be done by the gateway device by means of protocol-dependent software and applications. This causes the whole service provision chain to be subject to the specifications of the sensors employed, and also the incorporation of new devices that could enhance existing services or provide with new ones is restricted.

For example, in [44,64] they employ a set-top-box and a smartTV as gateways to connect different devices via Bluetooth, specific software and applications are needed in order to control each of the devices and services are built on top of those specific components. As such, in [64], they do not consider the integration of new devices as a means of enhancing the ecosystem functionalities, and in [44] they rely on third party downloaded applications to control new incorporated devices, but do not specify how these new functionalities would be incorporated in the ecosystem infrastructure. In [43,63], on the other hand, they developed dedicated equipment to act as a gateway for a set of specific sensing devices (called U-pillbox and iMedBox respectively), but new specific software extensions and controllers need to be developed in order to process and merge data from new incorporated devices.

The importance of employing a middleware approach in complex scenarios is a matter that has been widely addressed in the sensor-related literature [102,104,105,106]. In [102] they performed a survey focused on M2M Systems for mHealth and focused one of their main research goals on studying how the integration and convergence of different communication technologies is approached in those heterogeneous scenarios. They conclude that even at a lower communication level, sensor integration in health-related scenarios is still an open issue despite the considerable amount of work conducted in the field. As such, the need for using upper level abstraction mechanisms so that features such as correct data aggregation, heterogeneity, scalability, quality of service (QoS), etc. are guaranteed in care ecosystems becomes imperative.

Results described in Section 4.13 show that considered papers that describe sensor integration mainly refer to the use of any form of middleware, and that most of them follow a service oriented approach (see Figure 14). Service oriented middleware (SOM) is found for instance in [65], where devices are abstracted using XML-based language and transmitted using web services. They propose a framework made up of a set of sub-modules that are used to interface with domotic systems. The framework implements an abstraction layer that hides the underlying technologies of the integrated devices, permitting their interaction even if they are not natively interoperable. It also provides developers with a unique representation of the acquired data for all devices. In [65] they make use of the LinkSmart Middleware which was developed within the EU project called Hydra for Networked Embedded Systems. The middleware allows to incorporate heterogeneous physical devices into applications through web services in order to control any device, providing the upper service layers with a unfied and standard compliant message. In [56], they developed an eServices platform based on events and messages that allows sensors to trigger notification events to the eServices platform using different protocols (WIFI, Bluetooth Low Energy and Zigbee). In addition, sensors receive their thresholds at startup through the eServices platform.

Within the SOM approaches, the use of the OSGi framework is widely spread in the papers, as 50% of them make use of OSGi [107] for service delivery. OSGi is a Java based SOM that defines a set of specifications which enable components to hide their implementations from other components while communicating through services. A significant example can be found in [54], where they describe the universAAL platform which was developed under the 7th framework program by the European Commission, aiming at developing an open platform and reference specification for Ambient Assisted Living (AAL) solutions. The OSGi framework is employed as middleware to build the software infrastructure, hiding the heterogeneity of the employed sensors.

#### 5.3.4. Considered Training Actions

The importance of training actions, which include documentation, wikis, forums, etc. is of great importance in terms of technological development and is well known in technological fields like computer science [108]. In fact, it could be one of the main causes of success of open ecosystems, and even of greater importance in the closed ones.

In terms of care and assistance, it has been shown in many studies over the years that senior users and persons with any type of disability face serious problems when it comes to interacting with the technology [83,109,110], and that knowing how to use technological tools beforehand is needed in order to have continued support and confidence [5,111].

For that reason, training actions should be a major concern in order to overcome their reluctance to be part of any type of technological ecosystem. As stated in one of the reviewed papers which carried out piloted studies with real users [71]: “Due to relatively low computer literacy within the users group, training activities were to be repeated nearly on all occasions when the project team was visiting the Residential House.” However, obtained results from the reviewed papers show that only 10 papers considered them in any form, and that just six papers focus on training actions aimed to technology usage, and from those only three [21,44,71] are oriented to the patient end users. Thus, it is evident that the reviewed papers lack the consideration of training actions as one of the main axis for ecosystem success.

This issue has also been addressed by other authors in other ecosystem fields, not only in terms of end-user usability but also in terms of the easiness of developing new functionalities into the ecosystem. For example, in a recently published systematic mapping [112], they studied the requirements of software ecosystems engineering activities in papers ranging from 2009 to 2017 and found that just one study [113] out of 44 reviewed papers provided insight into software platform usability for platform developers and for third-party developers.

## 6. Threats to Validity

It has to be noted that, as with any research method, there could be threats to its validity and limitations in the current systematic review. The first threat is that the inclusion of all the relevant projects in the field of ecosystems in care and assistance is not guaranteed. This threat was mitigated by piloting different searches and combining different databases and manual searches. However, as can be depicted from the results, the number of papers that have reached the final stage after applying the quality criteria is quite low if compared with the number of papers retrieved from the search. The main reason for this is that, even though we have developed specific tools for aiding in the search string construction, the research field of technological ecosystems in care and assistance is an emerging field, so scarce information can be retrieved. For that reason, the search scope was broadened so as to capture as much information as possible within the limits of the review scope, and tools for fast abstract screening have also been developed for that purpose as described in Section 3.4.

A limitation of the proposed methodology and tools relies in employing only the retrieved abstract from each study. As the abstracts of publications contain way less information than is contained in the full paper, this could lead to a bias in the search results. In addition, as there could be different configurations of the parameters involved in the proposed stages (e.g., the minimum document frequency for a term to be considered, the stop words to be excluded, etc.) the set of parameters could introduce new threats to validity. It has to be noted, however, that the employed methodology for search string construction and abstract screening is not intended to fully automate the process of document search and retrieval, but to be combined with the researchers’ knowledge of the field. These stages were peer-reviewed so as to ensure the fulfilment of the research goals, and searches were first piloted to ensure that relevant terms were employed that produced enough results related to the topic of interest.

There are also two threats to internal validity in this systematic review. The first threat is that, due to the wide scope of the ecosystems described in the papers that include not only many different technologies, layers and components (such as business models or actors value propositions), most of the papers do not provide detailed descriptions or references for all the research and mapping questions. The second threat is related to the identification of values for classification criteria which again was not obvious in many cases, as many papers contained vague or fuzzy descriptions for the grouping terms employed for data extraction. In this case, some strategies (such as the quality assurance checklist) were used to mitigate this effect. In addition, the different resources provided (such as the public spreadsheets (http://bit.ly/2Pjo7bQ) that contain the results from databases as well as the results of the different stages of the review process were intended to reduce or remove any kind of bias, and also to make the whole process reproducible for the reviewers and readers.

## 7. Challenges, Gaps and Opportunities

The aim of this section is to add a more critical discussion regarding the research gaps, challenges and opportunities that can be extracted from the revised literature.

Taking into account the propositions of added value found in the proposals, the opportunities of delivering care innovations through technological ecosystems include:

- Remote access to the relevant care records. Doctors have quick and direct access to patient records, which they can share quickly and safely.
- More effective and assisted clinical decision making. Health authorities and institutions have the possibility of evaluating the effectiveness in assistance provision, allowing them to take decisions in the care provision systems. The interconection of health and care staff also makes it possible for people to receive more personalized and precise care services—reducing errors.
- Real-time monitoring of structured patient care delivery. Which allows a more personalized attention for example via portals that make it possible for people to browse through the available doctors and carers, read patient feedback and book their next doctor’s appointment, thus improving quality and providing easier access to medical facilities. The evolution and adoption of new and cheaper sensor technology allows to better provision home monitoring systems and integrate new services.
- Opportunity for patient (and carers) participation in the care process. Increasing the communication amongst different ecosystem actors not only allows doctors, hospitals and care institutions to be more accessible to patients, but also helps patients to connect with other patients. Furthermore, it allows patients and carers to be aware of treatment options along with preventive measures.
- Stats and management information which are a product of the care process. The information extracted from the ecosystem information exchange (taking into account privacy and security issues) allows the establishment of new business models that could attract other stakeholders even not directly related to the care provision.

However, results show that there are still many challenges and gaps that need to be fulfilled for developing technological ecosystems in care and assistance mature enough to provide the above opportunities in a consistent way. The ideal would be to follow a path to a single solution set, evolving from innovative solutions into a single ecosystem solution that involves all the ecosystem stakeholders. In this sense, one of the main gaps in the reviewed literature lies the maturity of the solutions found. As stated before, this could be related to the delay in indexing new developments in the selected sources. In any case, as a result of this lack of maturity there exist numerous ongoing initiatives for example at a European level such as the projects ACTIVAGE, Sofia2, SeniorSome, OneM2M, BigIOT, or MONICA focused on developing ecosystems in the health and care sector. The fact that they have not been found in the current search may be due to the fact that some of the proposals are either ongoing projects, they are not specific to the care sector or they do not have published research papers in relevant databases, but are rather found through communities and fora. Nevertheless, the existence of these initiatives denotes there is still much work to be done in the field.

Focusing on the obtained results, from the point of view of the software and technology structure there is still a great margin of development. For example, in terms of the openness of the proposals to include new software enhancements, only 5% of the reviewed papers provide means to modify the existing software. Including new software developments not only benefits from the point of view of ecosystem openness and expandability of the platform but also allows the inclusion of third-party software developers into the value chain. The fact that cornerstone and infrastructure based ecosystem architectures are the main types of ecosystems found makes the governance of the ecosystems highly centralized. This causes the existing software technologies, although modular and able to accept the inclusion of new software, in many cases to not be specifically designed for a care and assistance environment, and it is difficult to take into account aspects such as the privacy of the data or the interoperability between medical information systems. This results in a general lack of means to model and standardize the inclusion of new software developments, again hindering the incorporation of new potential stakeholders. In the same sense, although many proposals make reference to the possibilities of new device integration through different kinds of middleware, few of them take into account medical standards for connecting new sensors and medical devices. Not considering medical standards makes potential medical device developers back down when it comes to being part of the ecosystem.

It seems evident that the above gaps have a direct influence on the ecosystem business model. In this sense, the business structure is generally omitted in the found literature, along with the lack of involvement of the potential stakeholders in the development of the ecosystem since the first stages of technological development. In general terms, the approach taken to provide services is to make measurements of clinical information or environmental monitoring in the patient’s home and then share it through a centralized platform in which doctors or caregivers can make decisions about care provision. This user-centric model, rather than stakeholder-centric, makes it complicated to develop business models once the platform has been implemented and to attract other ecosystem actors. In this way, business models are reduced to patients and families care providers (individuals or organizations) using the ecosystem services. However, scarce attention is given to other business models such as: IT support and maintenance, providing training for end users or third party developers, develop ecosystem data and analysis tools (such as dashboards) and even to third parties incorporating or publishing other non-care-related services such as amenities for the dependant end users.

Due to the above, there is also a lack of an organizational structure in order to govern the ecosystem proposals, which in fact has a negative impact when it comes to attracting health authorities and governmental organizations. Although the process of delivering care is quite generic, and its complexity varies due to different factors such as geography, different patients health conditions, etc. found ecosystems rarely take into account this variability from an organizational perspective. To achieve this claim, platforms should feature a high degree of modularity while using established standards, and also consider other aspects such as data ownership, security, privacy etc., which in many cases vary depending on the country or even region. Although some of the found proposals take these issues into account, they lack the organizational structure necessary to apply them in practice. Thus, they do not provide the necessary links between the software and business structures, and also they lack an adequate organizational structure to govern the different technological solutions from a global (regional, national or international) perspective.

All the above gaps can be seen as opportunities for developing future lines of research. To understand and model care providing ecosystems, different paradigms still need to be applied in order to capture a more realistic view, bridging the gap between the ecosystem model and the ecosystem that needs modelling. For example, in terms of establishing well founded software and business organizational links, more effort should be made in characterizing the different ecosystem components from the very beginning. As such, incorporating the organizational and business structures during meta-modelling the ecosystem software structure and taking them into account in the data ontologies could help to reduce these gaps. An example of efforts in this direction can be found in [114]. On the other hand, more work is needed in the definition of clear and formal guidelines to develop this type of ecosystem, where the different stages to be carried out are identified and clearly specified (see for example the work of [21]). These guidelines should include the study of the models of health provision and incorporate the governmental organizations from the beginning of the development, so that the policy and legislation of the different areas are taken into account to develop cross-cutting platforms that would allow participants to interact similarly to those in other industries (e.g., as it happens in software ecosystems). Facets of policy and the relationship between the carer, the user and the authorities which works to create a formalised care giving situation should be included. Many examples of works in this direction can be found in the different European research projects and initiatives [115]. In addition, more research is needed in order to develop tools to evaluate the performance of the ecosystems with specific metrics that take into account not only medical and home monitoring data, but also user satisfaction of the different actors involved. Furthermore, means to make this data available for third parties should be studied (taking into account interoperability, security and privacy) so that they can exploit them and increase the business model and the value chain. Finally, incorporating and standardizing training actions for care provision, adding new services into the ecosystem, new devices, new software and instructions on how to use all the available tools, should be approached in order to attract not only end users but also third party developers.

## 8. Conclusions

In this paper we have presented a systematic review and mapping of the literature to identify, analyze and classify the published research carried out to provide care and assistance services from a technological ecosystems’ perspective. The research was conducted using systematic review and mapping in accordance with the guidelines proposed by Kitchenham and other authors in the field of software engineering. After applying the developed systematic search and review protocol, 37 papers that described technological ecosystems in care and assistance were obtained, data was extracted and results were analysed to give answers to our main research questions: how are these types of ecosystems considered in the literature, what are their distinctive characteristics from technological, care and assistance points of view. The main findings obtained from this process can be surveyed as:

- There is not a single concrete definition that encompasses all the characteristics of a care and assistance ecosystem. However, most definitions share common characteristics in terms of providing technological means to a community of users (patients, relatives, carers, doctors, etc.) in order to involve all of them in providing better care and assistance related services.
- The field of technological ecosystems in care and assistance is indeed an emerging field, as most publications found date from 2011 onwards.
- Most of the papers’ main efforts are focused on developing ecosystems where actors interact on top of a common technological platform, either developed from scratch or adapted from existing infrastructures and where the governance is usually centralized.
- The maturity of the ecosystems found in the literature is mainly conceptual, however, some of these conceptualizations have been evolved to concrete solutions.
- Ecosystems are usually user-centred rather than stakeholder-centred, where aggregated services and architectures are mainly focused on the patient end users. Few ecosystems take stakeholders into account during the designing stage.
- Employed devices for providing services are of a very diverse nature depending on the intended services, but many of them do not take into account medical standards.
- As platform or infrastructure centred ecosystems, the degree of the ecosystems’ openness to new devices greatly depends on the followed methodology for device integration within the service chain. Service oriented middleware (SOM) is the preferred approach in the studied papers.
- There is a lack of training actions considered in order to facilitate or even create the relationship between the different ecosystem actors and the underlying technological components.

## Figures and Tables

**Table 1:** Examples of pilot searches on the Scopus database and obtained results (as for May–2018).

Search String	Number of Results Obtained
ecosystem AND older person	7
ecosystem AND elderly	258
ecosystem AND (elderly OR care)	3997
(“digital ecosystem” OR “software ecosystem” OR “technological ecosystem”) AND (elderly OR care)	5

**Table 2:** Extracts of the selected prototype abstracts as prototypes for similarity computation.

Abstract 1	Effective provision of care and assistance services in ambient assisted living requires the involvement and collaboration of multiple stakeholders. To support such collaboration, the development of an ecosystem of products and services for active aging plays an important role. This article introduces a conceptual architecture that supports such care ecosystem. In order to facilitate understanding and better interrelate concepts, a 3-layered model is adopted …
Abstract 2	Assistance technologies are directly linked to the elderly and the disabled people. The necessary services offered by an assistance technologies ecosystem are provided mostly through internet. It is also well known that elderly and disabled people find it difficult to access the internet. The resulting challenge is to discover a combination of assistance technologies, meaning devices and software interface characteristics in order to give the opportunity to elderly and disabled to access the internet and enhance their autonomy to the extent made possible by electronic services. To address this, we elaborate on the concept of ecosystems to propose the notion …
Abstract 3	Patient-centric healthcare and evidence-based medicine with the emphasis on prevention and wellness promise to deliver better and more affordable healthcare. At minimal, they require health related information to be shared among a community including patients, providers, payers, and regulators. It is important for IT systems to facilitate information sharing within such … In particular, the concept of shared infrastructure and services provides the foundation for supporting healthcare service ecosystems. This paper proposes an ecosystem approach to identify …

**Table 3:** Quality assessment checklist.

Question	Score
Does the proposed solution apply to the whole technological ecosystem? (or describes it partially i.e., the software, business, etc. structure?)	Yes/Partially/No
Is the ecosystem architecture clearly described and defined?	Yes/Partially/No
Are the main goals of the ecosystem reported?	Yes/Partially/No
Are the ecosystem actors clearly identified and their roles specified?	Yes/Partially/No
Does the paper state which technologies are employed and how they are integrated into the ecosystem?	Yes/Partially/No
Are the services provided within the ecosystem adequately described?	Yes/Partially/No
Is the ecosystem implemented in the real world? (I.e., What is the maturity of the proposed solution? Is it a proof of concept, a piloted prototype architecture or a real implemented solution.)	Yes/Partially/No
Does the paper outline the methods used to evaluate the performance of the ecosystem as well as the results of that evaluation?	Yes/Partially/No
Are all research questions answered adequately?	Yes/Partially/No

**Table 4:** Summary of the followed steps in our protocol.

Step	Papers Retrieved	Percentage of Papers Left
Execution of the query string in the databases	23,797 (11,850 Scopus), (11,947 WoS)	100%
Remove duplicate studies and apply the cosine similarity threshold	809	3.4%
Apply the rest of inclusion criteria reviewing titles and abstracts, and full text when necessary	194	0.8%
Apply quality criteria based on article’s full text	35	0.15%
Remove instances of the same work (3 papers removed)	32	0.13%
Review papers primary references (5 papers added)	37	0.16%

**Table 5:** Related definitions of technological ecosystems in health and care in the studies.

Paper	Year	Definition
[10]	2004	“New technological developments, although not solving all problems, can be part of a new concept of integrated care system. An integrated elderly care system consists of a number of organisations such as care centres/day centres, healthcare institutions, and social security institutions, that involve the cooperation of a number of different human actors, e.g., social care assistants, healthcare professionals, elderly people, and their relatives. If based on computer networks and adequate supporting tools, the collaboration among the care institutions may evolve towards operating as a long-term virtual organisation and the various actors involved become part of a virtual community (VC).”
[49]	2011	“According to the definition, virtual communities can be used to support many processes in the elderly care sector. For example, they support the social interaction between clients and (informal) caregivers. ICT-based synchronous and asynchronous communication may expand client and (in)formal caregiver communication methods. VCs allow for the exchange of public and private information between involved parties and services. Profile- or context-based matchmaking allows for suggestions to find friends, activities and services. Reminder and agenda services can be moderated by and tailored to community members to increase and to organise socialisation. A VC can give personalised medication reminders and compliance rates when connected to medication registration. When mobility is integrated as in our case, ambulant telemonitoring services can be integrated for physiological signal monitoring and for feedback purposes.”
[48]	2013	“However, there is not a single concrete definition that encompasses all the characteristics of a health ecosystem. A definition encompassing the approaches of several research and commercial efforts (e.g., …) states that: A health ecosystem is defined as an environment where personal and public health related data are collected and distributed. Health care professionals are able to communicate with each other, apply appropriate treatment and avoid medical errors. Also, patients are continuously connected or online forming virtual communities.”
[21]	2014	“the interaction of a set of actors on top of a common technological platform that results in a number of software solutions or services … Further, Each actor is motivated by a set of interests or business models and connected to the rest of the actors and the ecosystem as a whole with symbiotic relationships, while, the technological platform is structured in a way that allows the involvement and contribution of the different actors.”
[46]	2014	“A Medical Eco-system essentially enriches medical practice with innovative technologies and systems but ensure interoperability, coordination, collaboration and synergy between physicians, patients and organizations since they comprise an integrated ecosystem.”
[43]	2015	“… the Health-IoT is a business over shared infrastructures including the internet backend facilities, core networks, access networks and mobile terminals… the health care service providers … should make use of the existing infrastructures owned by the internet content providers. Then the contents of Health-IoT services are delivered through the channels of telecom operators. On the other hand, the internet content providers and telecom operators should get the health care content from health care service providers instead of create it by themselves. The health care financial sources should encourage and protect such cooperation by financing the content providers in some way. The privacy regulations and public authentications should be applied to the content providers and telecom providers, as strictly as they are applied to the health care service providers.”
[44]	2017	“The concept of ecosystem of applications is proposed to refer to the technological infrastructure to support a collection of related services and, in the case of social and health care, to offer the communication and interaction mechanisms required to guarantee integrated care.”
[47]	2017	“draws from service-dominant logic (SDL) and underlines systemic value creation within a network of actors [23]. It emphasizes the contribution of all actors, … including patients and their families and friends, other patients, health care professionals, hospitals, health support agencies, professional associations, health insurers, health care authorities, government agencies, and regulatory bodies … SDL draws from the idea of togetherness; actors in the ecosystem use their knowledge and skills to provide benefits or value reciprocally to others and themselves”
[45]	2018	“In order to truly integrate hospitals or clinics with patients in PCC, there is a need to utilize the powerful ecosystem of IoT … IoT ecosystem is a convergence of sensors, actuators, telecommunication, cloud computing and big data, interconnecting them through the Inter net to provide goal-oriented services”. Where PCC is Patient Centred Care which is defined as: “Healthcare that establishes a partnership among practitioners, patients, and their families (when appropriate) to ensure that decisions respect patients’ wants, needs, and preferences and that patients have the education and support they need to make decisions and participate in their own care.”

**Table 6:** Publication sources.

Paper	Source
[6]	International Technology, Education and Development Conference
[7]	Computer Methods and Programs in Biomedicine
[8]	Proceedings of the 9th International Conference on Ubiquitous Information Management and Communication
[9]	International Multi-Conference on Systems, Signals and Devices
[10]	International Journal of Networking and Virtual Organisations
[11]	IEEE Transactions on Information Technology in Biomedicine
[21]	Inf. Sofw. Technol.
[48]	International Conference on Smart Homes and Health Telematics
[44]	Journal of Biomedical and Health Informatics
[52]	Informatics for Health and Social Care
[53]	IEEE International Conference on Digital Ecosystems and Technologies
[45]	Future Generation Computer Systems
[54]	Journal of intelligent systems
[55]	Proceedings of the IEEE
[56]	Sensors
[57]	Journal of Research and Practice in Information Technology
[58]	IEEE International Conference on Digital Ecosystems and Technologies
[59]	Proceedings of the 2016 Future Technologies Conference
[60]	Journal of Medical Systems
[61]	IEEE/ACM International Conference on Utility and Cloud Computing
[47]	Proceedings of the 21st International Academic Mindtrek Conference
[62]	IEEE International Conference on e-Health Networking, Application and Services
[63]	Wisdom Web of Things
[64]	International Conference on Information and Communication Technologies for Ageing Well and eHealth
[65]	Global Internet of Things Summit
[66]	Information Sciences
[67]	Future Visions on Biomedicine and Bioinformatics
[43]	Enterprise Information Systems
[68]	IEEE 18th International Conference on e-Health Networking, Applications and Services
[46]	International Conference on Management of Emergent Digital EcoSystems
[69]	IBM Journal of Research and Development
[70]	Innovative and Creative Developments in Multimodal Interaction Systems
[71]	International Journal of Medical Informatics
[72]	Journal of Ambient Intelligence and Smart Environments
[73]	Handbook of Ambient Intelligence and Smart Environments
[49]	International Journal of Networking and Virtual Organisations
[50]	European conference on artificial intelligence

**Table 7:** Authors’ names and number of publications.

Name	Total
Camarinha-Matos, LM	2
Awada, Imad Alex; Cramariuc, Oana; Mocanu, Irina; Seceleanu, Cristina; Kunnappilly, Ashalatha; Florea, Adina Magda; de Backere, F; Bonte, P; Verstichel, S; Ongenae, E; de Turck, F; Balasubramanian, V; Stranieri, A; Kaur, R; Berndt R.-D., Takenga M.C., Kuehn S., Preik P., Sommer G., Berndt S.; Camarinha-Matos, LM; Afsarmanesh, H; Chou, LD; Lai, NH; Chen, YW; Chang, YJ; Yang, JY; Huang, LF; Chiang, WL; Chiu, HY; Shin, HY; Christensen, HB; Hansen, KM; Kyng, M; Manikas, K; Christopoulou, S; Kotsilieris, T; Dimopoulou, N;Costa, CR; Anido-Rifon, LE; Fernandez-Iglesias, MJ; Eichelberg, M; Busching, F; Steen, EE; Helmer, A; Thiel, A; Hein, A; Wolf, L;Fabbricatore, C; Zucker, M; Ziganki, S; Karduck, AP; Farahani, B; Firouzi, F; Chang, V; Badaroglu, M; Constant, N; Mankodiya, K;Ferro, E.; Girolami, M.; Salvi, D.; Mayer, C.; Gorman, J.; Grguric, A.; Ram, R.; Sadat, R.; Giannoutakis, K.M.; Stocklöw, C.; Helal, S.; Chen, C.; Kim, E.; Bose, R.; Lee, C.; Marcelino, I; Laza, R; Domingues, P; Gomez-Meire, S; Fdez-Riverola, F; Pereira, A; Ibarz, A; Falco, JL; Vaquerizo, E; Lain, L; Artigas, JI; Roy, A; Kielland-Aanesen, HA; Borras, J; Kor, AL; Yanovsky, M; Pattinson, C; Kharchenko, V; Li, SH; Wang, CY; Lu, WH; Lin, YY; Yen, DC; Li, Y; Guo, L; Guo, Y; Litovuo, L; Makkonen, H; Aarikka-Stenroos, L; Luhtala, L; Makinen, S; Lu, SH; Lai, KC; Yang, DL; Tsai, MH; Li, KC; Chung, YC; Ma, JH; Yen, NY; Huang, RH; Zhao, X; Macis, S.; Loi, D.; Pani, D.; Rijnen, W.; Raffo, L.; Miori, V; Russo, D; Lamprinakos, G.C.; Asanin, S.; Broden, T.; Prestileo, A.; Fursse, J.; Papadopoulos, K.A.; Kaklamani, D.I.; Venieris, I.S.; Oberleitner, R; Reischl, U; Lacy, T; Goodwin, M; Spitalnick, JS; Pang, ZB; Zheng, LR; Tian, JZ; Kao-Walter, S; Dubrova, E; Chen, Q; Pecoraro, F; Luzi, D; Pourabbas, E; Ricci, FL; Qureshi, B; Ram, R; Peres, Y;Rosas, J; Camarinha-Matos, LM; Carvalho, G; Oliveira, AI; Ferrada, F; Stav, E; Walderhaug, S; Mikalsen, M; Hanke, S; Benc, I; Stefan, I; Aldea, CL; Nechifor, CS; Tazari, MR; Furfari, F; Ramos, JPL; Ferro, E; Van’t Klooster, JW; Van Beijnum, BJ; Pawar, P; Sikkel, K; Meertens, L; Hermens, H; Wolf, P; Schmidt, A; Klein, M;	1

**Table 8:** Examples of different services described in the papers.

Type of Service	Examples in the Papers
health or care	indicate caregiver location and select which caregiver is more suited to execute a task [7,11]; remote physiological transmission and management [52,53]; health records transmission and management [44,60]; etc.
environmental monitoring	generate alerts from house parameters [56,66,68]; health-related environmental monitoring [7,59]; fall prevention [6,52]; etc.
reminder and information	medication reminders [49,63,71]; schedule personal doctor appointments [64,65]; agenda reminders [10,57]; etc.
social interaction	social networks [44,64]; share experiences with doctors and caregivers [43,46,59,63]; leisure services [10,56] etc.
rehabilitation and maintenance	rehabilitation monitoring [44,52,62]; serious games [44,47,57]; etc.

**Table 9:** Methods employed for testing the ecosystem.

Paper	End Users’ Questionnaire Evaluation	Evaluate Running System Performance	Simulation	Evaluate Ecosystem Components
[11,56,67], [44,66,69], [71]	x
[8,45,60], [43,52,58], [57]		x
[62,70]			x
[7,61]				x
[72]	x	x
[6]	x			x

**Table 10:** Relation of health related standards cited in the studies.

Paper	HL7	XDS	ISO/IEEE 11073	ISO 13940	HIPAA	Contribute in the Creation of Standards	Propose Their Own Standard
[9,45,56,60,62]	x						
[21]	x	x					
[54]						x	
[58]							x
[66]	x		x				
[67]					x		
[68]				x			
[46,71]	x		x				

**Table 11:** Complementary training actions in the studies.

Paper	Health Education	Technology Usage	Software Development	Systems Integration	Training Action
[11]	x				Care volunteers are trained by care professionals
[21]	x	x	x		Guides, tutorials, and code support that allows non-expert users to produce valid clinical data. Online information resources for software development. Provide testing environment of new functionalities
[48]	x	x			Documentation to support editing, addition of new or removal of existing technological devices.
[44]	x	x			Demonstration of system usage in piloted environments. Content providing continuing education to caregivers.
[54]	x	x	x		Documentation for developers for adding new services. Training material for training new technical users. It includes documents, presentations, videos, and material for courses.
[58]		x	x	x	On-line documentation for software development and technology usage and standard compliance.
[60]	x				Remote health education training through courses on a Learning platform: Audio/video courses, Course inquiry, Learning platform, Course satisfaction survey …
[67]	x				Clinical training for doctors from an annotated video database of children with autism spectrum disorders.
[68]	x				Social community patient education for increased awareness of the problems so as to instill autonomy in the patient to take care of their own illness.
[71]		x			In home training activities on technology usage to the senior users.

**Table 12:** Care and assistance technological ecosystem concepts found in the revised literature.

Paper	Care and Assistance	Technology	Ecosystem
[10]		“computer networks and adequate supporting tools”	“involve the cooperation of a number of different human actors, e.g., social care assistants, healthcare professionals, elderly people, and their relatives”
[49]	“apply appropriate treatment and avoid medical errors”	“personal and public health related data are collected and distributed”	“Health care professionals … communicate with each other … Also, patients are continuously connected or on-line forming virtual communities”
[21]		“common technological platform … allows the involvement and contribution of the different actors”	“interaction of a set of actors … with symbiotic relationships”
[46]	“enriches medical practice”	“innovative technologies and systems”	“interoperability, coordination, collaboration and synergy between physicians, patients and organizations”
[43]	“the health care service providers … should make use of the existing infrastructures”	“shared infrastructures including the internet backend facilities, core networks, access networks and mobile terminals”	“should make use of the existing infrastructures owned by the internet content providers”
[44]	“social and health care”	“technological infrastructure”	“to offer the communication and interaction mechanisms required to guarantee integrated care”
[47]			“systemic value creation within a network of actors including patients and their families and friends, other patients, health care professionals, hospitals … actors in the ecosystem use their knowledge and skills to provide benefits or value reciprocally to others and themselves”
[45]	“Patient Centred Care … patients have the education and support they need to make decisions and participate in their own care”	“convergence of sensors, actuators, telecommunication, cloud computing and big data”	“integrate hospitals or clinics with patients … establishes a partnership among practitioners, patients, and their families”
